# Noninvasive closed-loop acoustic brain-computer interface for seizure control

**DOI:** 10.7150/thno.99820

**Published:** 2024-09-09

**Authors:** Junjie Zou, Houminji Chen, Xiaoyan Chen, Zhengrong Lin, Qihang Yang, Changjun Tie, Hong Wang, Lili Niu, Yanwu Guo, Hairong Zheng

**Affiliations:** 1 The National Key Clinic Specialty, The Engineering Technology Research Center of Education Ministry of China, Guangdong Provincial Key Laboratory on Brain Function Repair and Regeneration, Department of Neurosurgery, Zhujiang Hospital, Southern Medical University, Guangzhou 510282, China.; 2 Institute of Biomedical and Health Engineering, Shenzhen Institute of Advanced Technology, Chinese Academy of Sciences, Shenzhen 518055, China.; 3 Shenzhen College of Advanced Technology, University of Chinese Academy of Sciences, Shenzhen 518055, China.; 4 The Brain Cognition and Brain Disease Institute, Shenzhen-Hong Kong Institute of Brain Science, Shenzhen Institute of Advanced Technology, Chinese Academy of Sciences, Shenzhen 518055, China.

**Keywords:** acoustics, brain-computer interface, vagus nerve stimulation, epilepsy, brain disease

## Abstract

**Rationale:** The brain-computer interface (BCI) is core tasks in comprehensively understanding the brain, and is one of the most significant challenges in neuroscience. The development of novel non-invasive neuromodulation technique will drive major innovations and breakthroughs in the field of BCI.

**Methods:** We develop a new noninvasive closed-loop acoustic brain-computer interface (aBCI) for decoding the seizure onset based on the electroencephalography and triggering ultrasound stimulation of the vagus nerve to terminate seizures. Firstly, we create the aBCI system and decode the onset of seizure via a multi-level threshold model based on the analysis of wireless-collected electroencephalogram (EEG) signals recorded from above the hippocampus. Then, the different acoustic parameters induced acoustic radiation force were used to stimulate the vagus nerve in a rat model of epilepsy-induced by pentylenetetrazole. Finally, the results of epileptic EEG signal triggering ultrasound stimulation of the vagus nerve to control seizures. In addition, the mechanism of aBCI control seizures were investigated by real-time quantitative polymerase chain reaction (RT-qPCR).

**Results:** In a rat model of epilepsy, the aBCI system selectively actives mechanosensitive neurons in the nodose ganglion while suppressing neuronal excitability in the hippocampus and amygdala, and stops seizures rapidly upon ultrasound stimulation of the vagus nerve. Physical transection or chemical blockade of the vagus nerve pathway abolish the antiepileptic effects of aBCI. In addition, aBCI shows significant antiepileptic effects compared to conventional vagus nerve electrical stimulation in an acute experiment.

**Conclusions:** Closed-loop aBCI provides a novel, safe and effective tool for on-demand stimulation to treat abnormal neuronal discharges, opening the door to next generation non-invasive BCI.

## Introduction

Epilepsy is a disorder of brain networks that affects approximately 70 million patients worldwide 1. Neuromodulation techniques have been used for treatment of epileptic patients including deep brain stimulation (DBS) and vagus nerve stimulation (VNS) 2. DBS implants electrodes in the brain to modulate neural circuits through electrical stimulation 3. Open-loop DBS of anterior thalamic nucleus or central median nucleus could reduce seizure frequency and severity 4, 5. VNS modulates neuronal activity in the nucleus tractus solitarius (NTS) via upstream fibers of the vagus nerve and further modulates the function of the locus coeruleus (LC), the dorsal raphe nucleus (DRN), the hippocampus and amygdala through vagal pathways 6, 7. Clinical studies have shown that 1 year of open-loop VNS treatment can reduce seizure frequency by more than 50% 8-10. However, a surgical procedure is required to implant the VNS device 11.

Ultrasound, as a non-invasive neurostimulation technique, has shown antiepileptic effects in human brain tissues, rodents, primates, and patient with epilepsy 12-19. Yoo et al. have verified that ultrasound stimulation of the hippocampus controls epileptic discharges in rodents 20. Lin et al. have demonstrated that ultrasound stimulation could reduce abnormal discharges in brain tissues of patients with epilepsy by increasing inhibitory inputs 12. Zou et al. have indicated that ultrasound stimulation of epileptogenic zone can effectively inhibit epileptic discharges in a non-human primate model of epilepsy 14. More exciting, ultrasound stimulation of epileptogenic zone could safely and effectively reduce seizure frequency and interictal epileptic discharges in patients with epilepsy 15, 21.

Recently, the modulatory effect of ultrasound stimulation of the vagus nerve has drawn prominent attention. Recent studies have shown that open-loop ultrasound stimulation of the vagus nerve can improve inflammatory arthritis and restore glucose homoeostasis 22-24. The cell bodies of the vagus nerve are in the nodose ganglion (NG) 25. Mechanosensitive ion channels (MSCs) such as Piezo1 and Piezo2 were shown to be present in NG and involved in a variety of physiological activities 26-28. Studies have shown that ultrasound can modulate multiple MSCs including Piezo1 and Piezo2 29, 30. Thus, ultrasound stimulation of the vagus nerve has the potential for seizure management and avoids the risks caused by surgery, which is important for realizing non-invasive treatment of epilepsy.

In addition, epilepsy is a stochastic disorder, and a therapeutic strategy of "on-demand" seizure control has been proposed to overcome the adverse effects of continuous stimulation 31-36. Many studies have shown that close-loop systems can effectively control seizures by identifying seizures based on electroencephalogram (EEG) and triggering light, electricity, magnetism or ultrasound to stimulate the brain 36-39. Moreover, clinical study has indicated that closed-loop VNS by recognizing the heart rate changes can reduce seizure frequency 40. In addition, closed-loop ultrasound neuromodulation systems have been developed by detecting the EEG signals, and triggering the ultrasound stimulation of brain, which can effectively suppress seizures 38, 39. However, the antiepileptic effects of closed-loop ultrasound stimulation of the vagus nerve are still unclear.

Detection and prediction algorithms of seizures are the core of closed-loop neuromodulation system 41, 42. Beniczky et al. used a thresholding algorithm based on time and frequency domains in EEG had an average sensitivity of 91% for each patient 43. Temko et al. have explored multiple features with a machine learning technique for automatic seizure detection 44. They used Fast Fourier transform for feature extraction of EEG signals followed by support vector machine (SVM) to detect neonatal seizures. It was able to achieve an average detection rate of ~89% with one false seizure detection per hour. In focal epileptic seizure detection, a convolutional neural network (CNN) and long short-term memory (LSTM) were adopted in EEG data with an area under curve (AUC) of 0.94 and specificity of 99.9% 45. Our previous study, based on the instantaneous frequency and spectral entropy metrics features of EEG signals detected seizures using LSTM and bidirectional LSTM (BiLSTM) networks with average detection accuracies of 73.33% and 81.33%, respectively 39. These current studies based on offline epilepsy data provide a foundation for closed-loop real-time seizure detection. Thus, the scientific basis exists for closed-loop acoustic brain-computer interface (aBCI) treatment of epilepsy, if these disparate findings can be integrated.

Three barriers have prevented the closed-loop aBCI. First, there is a pressing need to focus accurate and real-time algorithms on seizure onset detection based on EEG data, which do give their increasing importance in closed-loop neuromodulation for the treatment of epilepsy. Second, although electrical stimulation of the vagus nerve can treat epilepsy, no studies have proved that ultrasound stimulation of vagus nerve can control the seizures, including the effective stimulus parameters and modulation mechanisms. Third, the lack of closed-loop aBCI system hinder the noninvasive BCI to treat brain disorders such as epilepsy.

Here, we developed a new closed-loop acoustic brain-computer interface (aBCI) system, which decodes seizure events in real time and responsively triggers ultrasound stimulation of the vagus nerve to control seizures. Firstly, we established a multilevel threshold model as a decoder for closed-loop aBCI, which can automatically process EEG signals and detect seizures in real time, achieving on-demand seizure suppression to avoid overtreatment and improve safety. Then, we identify a parameter set that selectively activates MSCs in the NG while inhibiting neuronal excitability in the hippocampus and amygdala. Physical transection or chemical blockade of the vagal pathway abolishes the protective effect of aBCI on epilepsy. Collectively, these results demonstrate that the closed-loop aBCI provide a new tool for treatment of epilepsy.

## Results

### Non-invasive closed-loop aBCI system

**Figure 1:**
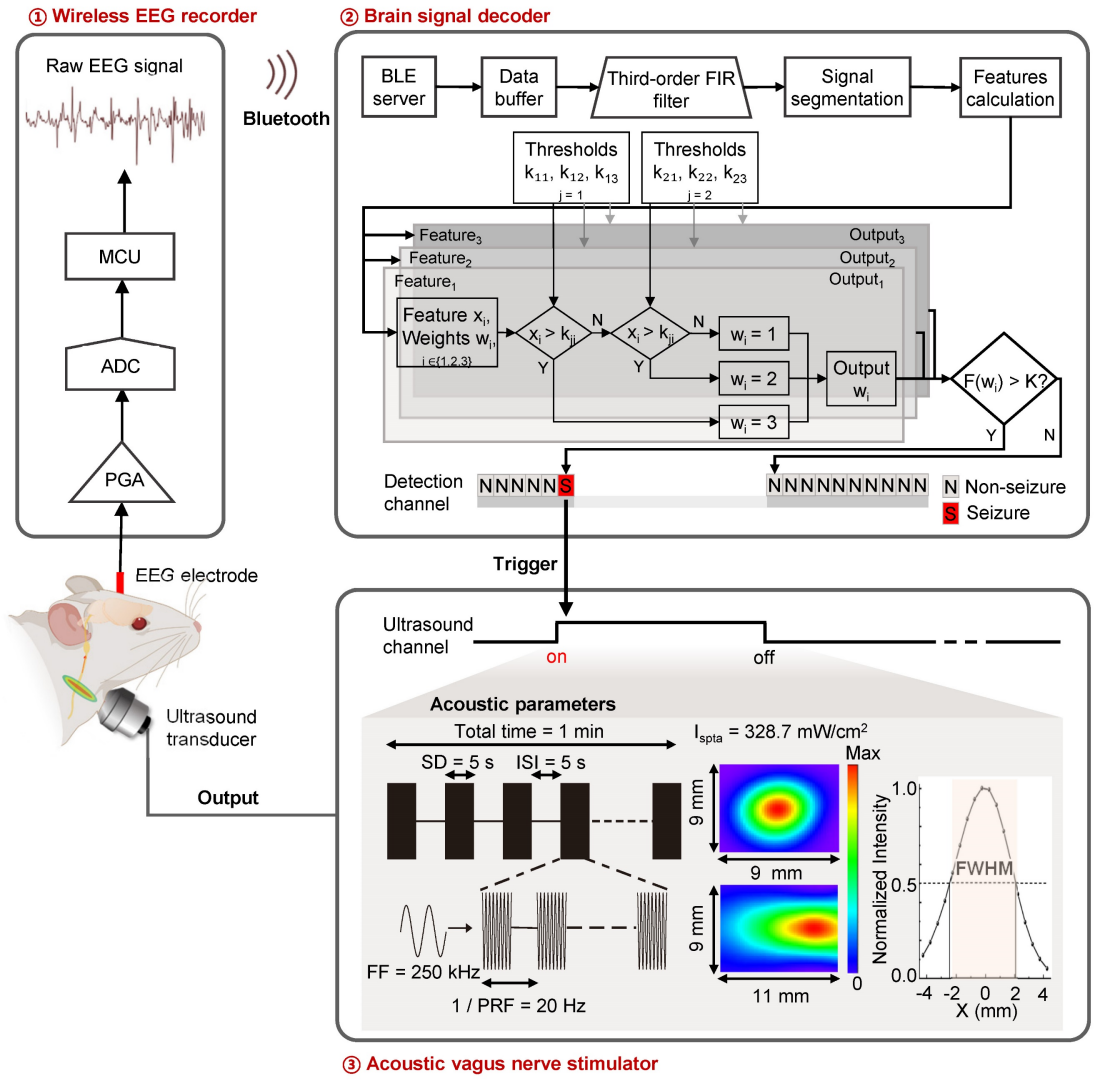
** Non-invasive closed-loop acoustic brain-computer interface system.** Electroencephalography (EEG) signals are recorded wirelessly and transmitted to the decoding software. The EEG data is pre-processed and divided into segments that are passed to an EEG signal decoder based on a multi-level threshold model for decoding. The decoder classifies the EEG signals into non-seizure and seizure status. When the decoder determines that the real-time EEG segment is seizure, a stimulator is triggered to stimulate the vagus nerve with ultrasound. The parameters of the acoustic vagus nerve stimulator including fundamental frequency (FF), sonication duration (SD), inter-stimulus interval (ISI), pulse repetition frequency (PRF), spatial peak temporal average intensity (I_spta_), full width at half maximum (FWHM).

To achieve on-demand vagus nerve stimulation to control seizure, we developed a non-invasive closed-loop aBCI system (Figure 1). The system consists of a wireless EEG recorder, a brain signal decoder and an acoustic vagus nerve stimulator. A recorder is fixed to the skull for monitoring electroencephalogram signals, which were wirelessly transmitted to the brain signal decoder for detecting seizure onset, then triggering the acoustic vagus nerve stimulator. The 9.4T magnetic resonance imaging (MRI) system (uMR 930, United Imaging) and high-frequency ultrasound imaging system with a 40 MHz ultrasound transducer (Vevo 2100, VisualSonics) were used to localize the cervical vagus nerve (Figure S1).

### Design of a real-time multi-level threshold model to detect seizures

**Figure 2:**
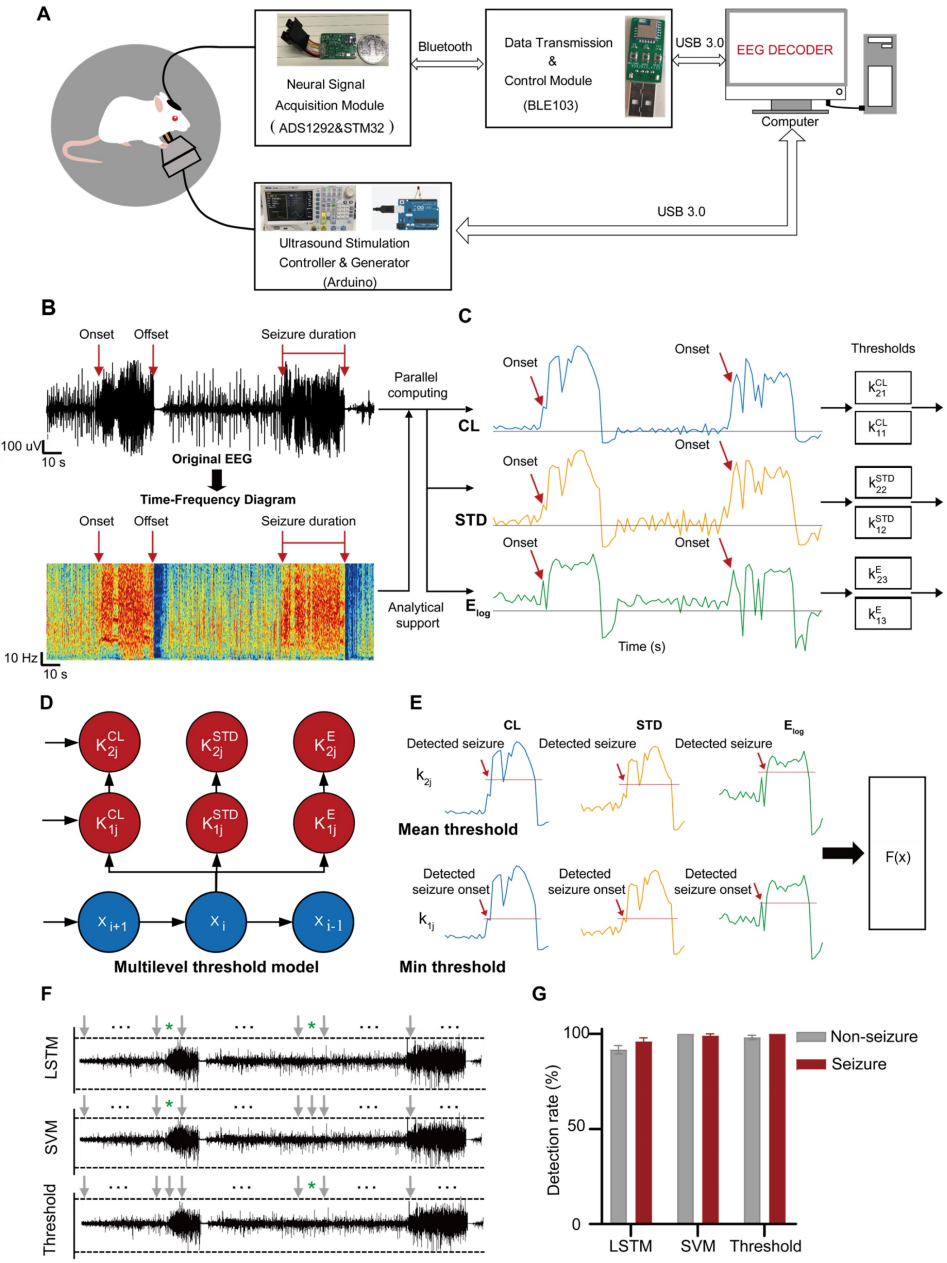
** The EEG signal decoder for seizure detection. A** Block diagram of an EEG-based closed-loop system for ultrasound treatment of epilepsy. **B** Raw EEG signals and time-frequency diagram containing two epileptic seizures. Onset represents the beginning of a seizure. Offset means the end of a seizure. Seizure duration is defined from onset to offset. **C** Filtered EEG signals were processed to compute three EEG features for the channel: {CL, STD, E_log_}. Seizure and non-seizure EEGs of six rats were used to calculate these three features, and the minimum of the statistical episode were used as the first stage threshold: {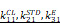} and the mean as the second stage threshold: {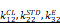}, where the temporal window size is 3s. **D** Schematic diagram of the multi-level threshold model used to determine whether each x_i_ is a seizure, where the index i denotes the i-th temporal window (bin size of 3s). **E** Illustration of a multilevel threshold decoding strategy for seizures. Firstly, the first and second stage thresholds are statistically derived from the previous EEG data. Next, we acquired real-time EEG signals and calculated the three features of CL, STD, and E_log_ based on a 3 s window. The size of the real-time feature was compared with the set first and second stage thresholds, and the ultrasound stimulation was triggered if all three feature values reached the first stage or two of them reached the second stage. **F** Demonstration of continuous online seizure detection in a sample recording session. Gray arrows denote true detection, and green stars denote false detection. **G** Comparison of seizure and non-seizure detection rates based on EEG decoding strategies using LSTM, SVM and multi-level threshold models. The detection rate is the average of five tests for each model.

As illustrated in Figure 2A, the circuit system of the aBCI consists of a neural signal acquisition module, a wireless data transmission and control module, a software interface module for real-time display, storage and playback of historical signals, and an ultrasound stimulation controller and an ultrasound signal generator. Online real-time seizure detection algorithms play a key role in the closed-loop aBCI, which determines when the closed-loop aBCI can give ultrasound stimulation. EEG is the most common source of signals in seizure detection. As shown in Figure 2B, abnormal EEG signals usually appear earlier than behavioral manifestations during a seizure, and abnormalities in the time-frequency map of seizure EEG signals can be clearly seen by time-frequency analysis, so the detection of the starting point of abnormal EEG signals can lead to more precise regulation for the closed-loop aBCI. We used pentylenetetrazole (PTZ)-induced epilepsy model in rats, and the EEG signals were filtered, analyzed, feature-extracted, model trained and classified into non-seizure and seizure states (Figure S2A). We acquired 3000 data samples and calculated the mean instantaneous frequency, mean spectral entropy, Euclidean norm, coastline (CL), standard deviation (STD), and logarithm of energy entropy (E_log_) to statistically analyze the EEG features of state seizures and non-seizures. Among these six features, there were significant differences between seizure features and non-seizure features (Figure S2B-G). According to the difference of means between seizure and non-seizure, we selected the CL, STD, and E_log_, and statistically obtained the thresholds for epileptic detection from the pre-collected EEG signal data (Figure 2C). We defined the minimum of these three features in the epilepsy samples as their corresponding first-stage thresholds, and their mean as the corresponding second-stage thresholds, and then apply the thresholds to the multilevel thresholding model (Figure 2D). In the real-time closed-loop aBCI system, the parallel calculation of features method is used, when three features in the channel are greater than the first stage threshold or two of them are greater than the second stage threshold, the multilevel threshold model considers that there is a seizure in the channel, and it will trigger the ultrasound stimulation control module on to achieve fast and reliable detection of seizures (Figure 2E).

In addition, we also trained LSTM and SVM with the same datasets and tested them for comparing the performance. The results showed that LSTM model had an average accuracy, sensitivity and specificity of 93.5%, 91% and 96%, respectively. SVM model reached an average accuracy, sensitivity and specificity of 99.5%, 100% and 99%, respectively. Multi-level thresholding model achieved an average accuracy, sensitivity and specificity of 99%, 98% and 100%, respectively (Figure 2F). Using these three decoders, we performed online aBCI experiments to detect continuously and automatically the onset of epileptic signals (Figure 2F-G). The results showed that the multi-level threshold model with less detection time and higher robustness, was used in the closed-loop aBCI system for seizure detection.

### Specific acoustic parameters for seizure control

**Figure 3:**
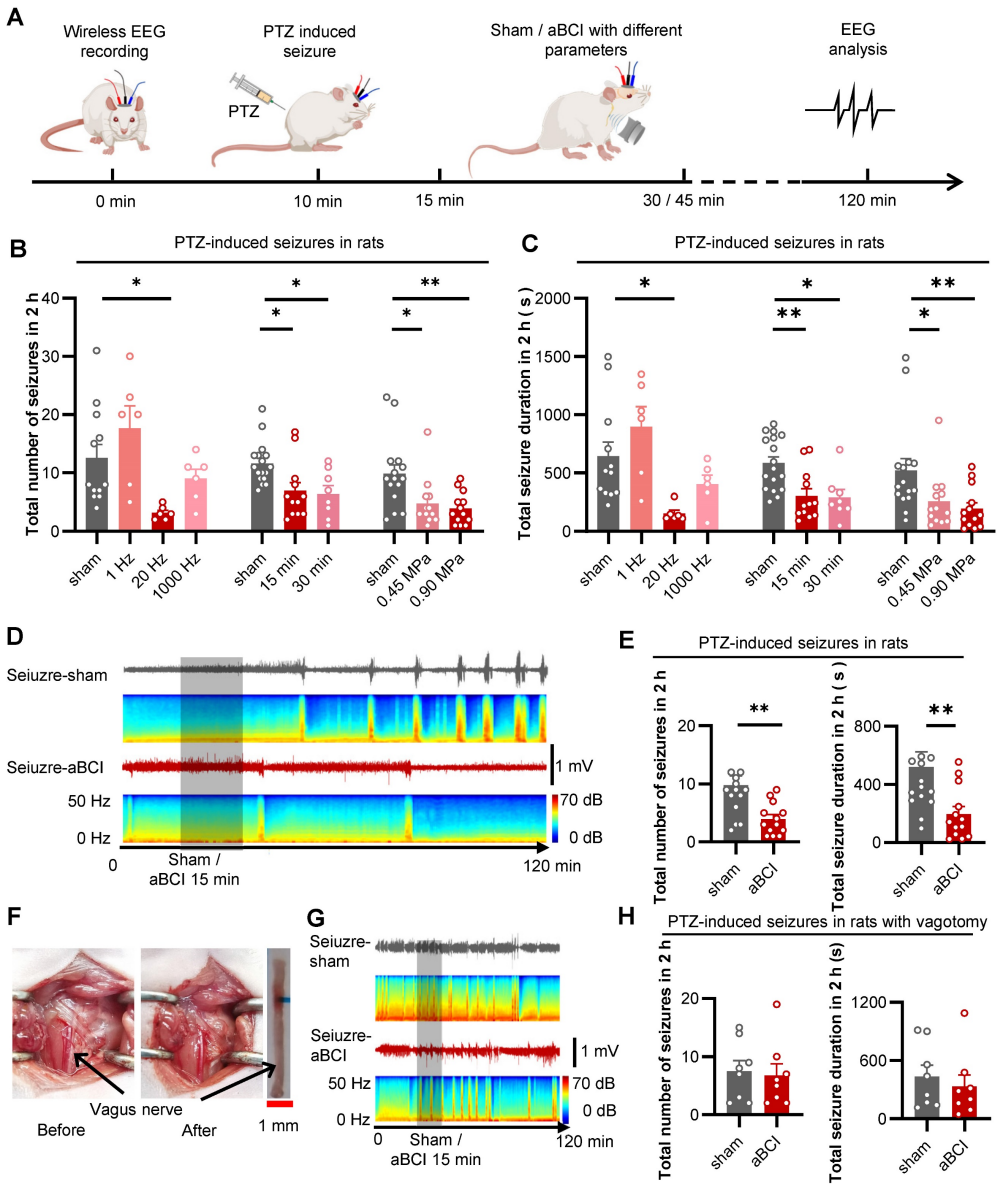
** Effective acoustic parameters selection for treatment of PTZ-induced seizures. A** The experimental timeline and procedures of different acoustic parameters. **B** Statistical graphs of total number of seizures within 2 hours for aBCI (open-loop aBCI) under different acoustic parameters. 20 Hz PRF aBCI effectively reduced total number of seizures, while 1 Hz trended to enhance seizure and 1000 Hz showed limited effect (sham: n = 12 rats; 1, 20, 1000 Hz: n = 6 rats each group). Both 15 min and 30 min insonation reduced seizure numbers, there was no significant different between the two insonation times (sham: n = 17 rats; 15 min: n = 12 rats; 30 min: n = 8 rats). In the acoustic pressure screening study, both 0.45 and 0.90 MPa aBCI showed effectiveness on reducing total seizure number and 0.90 MPa has slightly better effect (sham: n = 15 rats; 0.45, 0.90 MPa: n = 13 rats each). **C** Statistical graphs of total seizure duration for aBCI under different acoustic parameters. 20 Hz PRF effectively reduced total seizure duration, while 1 Hz trended to prolong the total seizure duration and 1000 Hz showed limited effect (sham: n = 12 rats; 1, 20, 1000 Hz: n = 6 rats each). Both 15 min and 30 min insonation time shortened total seizure duration but 15 min even better (sham: n = 17 rats; 15 min: n = 12 rats; 30 min: n = 8 rats). Both 0.45 and 0.90 MPa showed effectiveness and 0.90 MPa has better effect in controlling total seizure duration (sham: n = 15 rats; 0.45, 0.90 MPa: n = 13 rats each group). **B** and **C** *p < 0.05, **p < 0.01 by Dunns multiple comparisons test following Kruskal-Wallis test. **D** Representative EEG in the PTZ-aBCI and PTZ-sham groups. Sham or aBCI 15 minutes (show). **E** Statistical graphs of total number of seizures and seizure duration in two groups (sham: n = 15 rats; aBCI: n = 13 rats). The aBCI significantly reduced the total number of seizures induced by PTZ in 2 hours; Compare with the sham group, aBCI decreased the induced total seizure duration. **F** Photographs before and after vagotomy. **G** Representative EEG in the PTZ-aBCI and PTZ-sham groups with vagotomy, sham or 15 minutes aBCI (shadow). **H** Statistical graphs of total number of seizures and seizure duration for sham and aBCI in vagotomy rats (sham: n = 8 rats; aBCI: n = 8 rats). In vagotomy rats, aBCI cannot effectively reduce the total number of seizures; also, aBCI cannot control the total seizure duration. **E** to **H** **p < 0.01 by Mann-Whitney test. Data are presented as mean ± SEM.

To investigate the antiepileptic effect of aBCI on the vagus nerve, we used PTZ to induce acute seizures in rats. The antiepileptic effect of aBCI was investigated by analyzing the number of seizure events and total seizure duration in the EEG within two hours after PTZ injection. The acoustic parameters are crucial for seizure control. We tested different acoustic parameters including pulse repetition frequency (PRF), insonation time, and acoustic pressure to stimulate the vagus nerve to obtain optimal intervention parameters in epileptic rats (Figure 3A).

Firstly, different PRFs (1 Hz, 20 Hz and 1000 Hz) were used to explore the antiepileptic effect of aBCI (Figure 3B, C). The results showed that aBCI with 20 Hz PRF reduced the total number of seizures by more than 50% compared to the sham group, while 1 Hz PRF tended to increase the total number of seizures and 1000 Hz PRF had a limited effect. Meanwhile, 20 Hz PRF aBCI effectively reduced total duration of seizures.

Next, we studied the effect of insonation time (15 min and 30 min) on the antiepileptic effect (Figure 3B, C). The results indicated that both 15 and 30 minutes insonation time reduced the total number of seizures within 2 hours. Also, both 15 and 30 min aBCI decreased the total duration of seizures. There was no significant difference between the two insonation parameters, thus we chose 15 min insonation time combining 20 Hz PRF for the next acoustic pressure study.

Next, the effect of acoustic pressure (0.45 MPa and 0.9 MPa) on the antiepileptic effect was examined (Figure 3B, C). The results indicated that both interventions, with acoustic pressures of 0.45 MPa and 0.90 MPa, reduced the total number of seizures within 2 hours, while the aBCI intervention with 0.90 MPa demonstrated a slight superiority in efficacy. The same result appeared in total seizure duration. Although there was no significant difference between the two ultrasound stimulation groups, the 0.9 MPa group showed better control of both the total number of seizures and the total seizure duration compared to the 0.45 MPa group. Thus, we preferred 0.90 MPa for more reliable results. Therefore, we chose 20 Hz PRF, 15 min insonation time and 0.90 MPa acoustic pressure as the optimized parameters for the following study. The EEG results showed that aBCI with optimized parameters had a 65% reduction in the total number of seizures and a 67% reduction in total seizure duration compared to the sham group (Figure 3D, E).

Furthermore, we investigated the role of vagus nerve in the ultrasound antiepileptic effect by removing the left vagus nerve in rats (Figure 3F). The results showed no significant difference between aBCI and sham group in the total number of seizures and total total seizure duration (Figure 3G, H). These results suggest that the vagus nerve is essential for the antiepileptic effect of aBCI.

Moreover, we compared the acute antiepileptic effects of VNS and aBCI, (Figure S3A-C) and found that the total number and duration of seizures were significantly reduced in the aBCI groups. However, there were no significant differences in the total number of seizure and seizure duration in VNS group (Figure S3D-F).

### Antiepileptic effect and mechanism of the closed-loop aBCI

To examine the antiepileptic effect of closed-loop aBCI, we transferred the combination of acoustic parameters obtained in the previous study to the closed-loop aBCI system (Table 1) and a rat model of acute seizure was established by PTZ. The closed-loop aBCI system detected seizures within 30 minutes after PTZ injection and triggered ultrasound stimulation of the vagus nerve. The seizure duration was quantified based on EEG to evaluate the antiepileptic effect of closed-loop aBCI.

**Figure 4:**
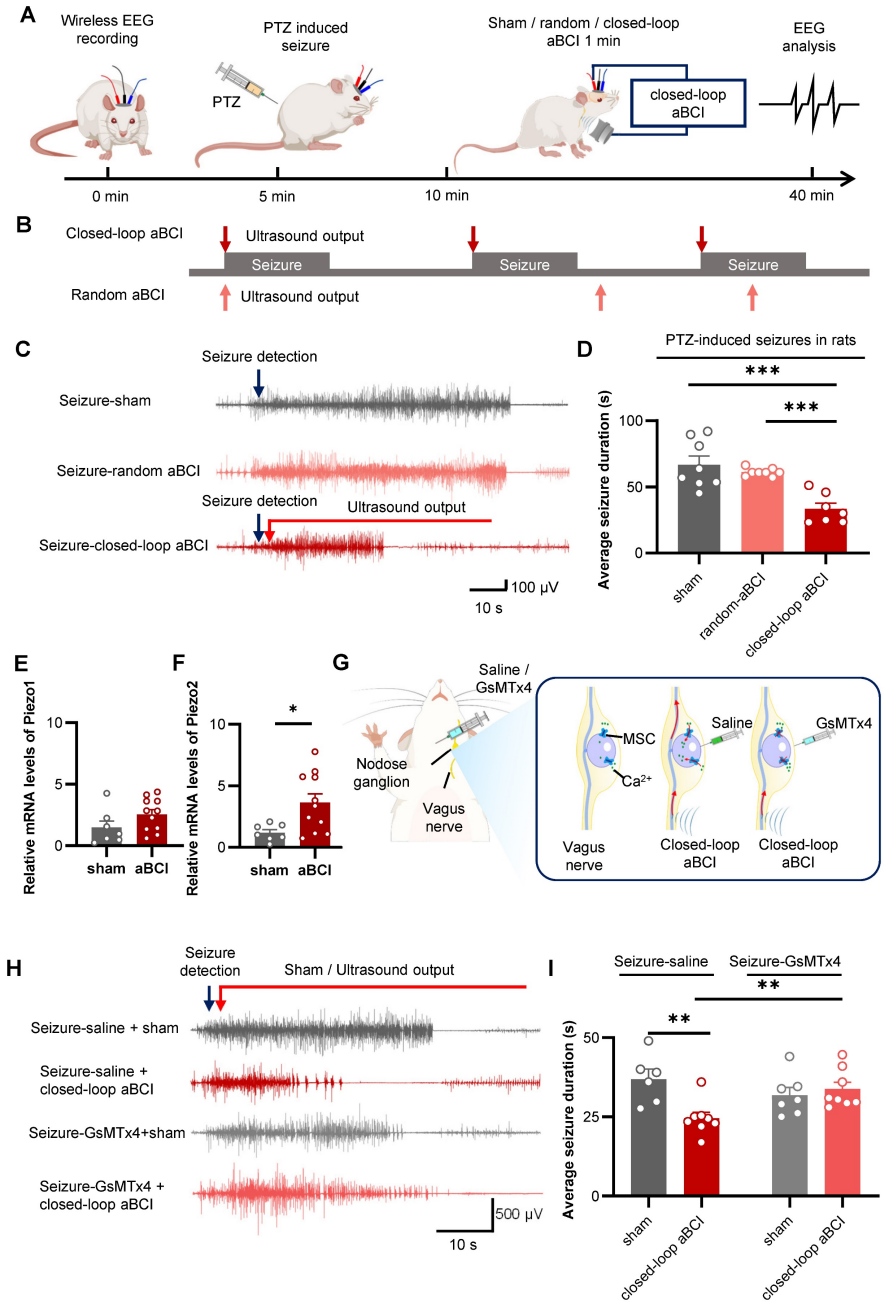
** Closed-loop aBCI system can effectively control seizures. A** The experimental procedures. **B** Schematic diagram of closed-loop aBCI and random aBCI. For closed-loop aBCI, the ultrasound output was triggered at the same time as the seizure was detected. For random aBCI, it was not controlled by seizure status and the ultrasound output was triggered randomly within 30 minutes after PTZ injection. **C** Representative EEG of sham, random aBCI and closed-loop aBCI. Seizure detected (blue arrow) and ultrasound output (red arrow). **D** Statistical graphs of average seizure duration for sham, random aBCI, and closed-loop aBCI. Closed-loop aBCI suppressed seizure duration while random aBCI stimulation failed to suppressed seizure duration (sham: n = 8 rats; random aBCI: n = 12 rats; closed-loop aBCI: n = 7 rats). ***p < 0.001 by Tukey's multiple comparisons test following one-way ANOVA. **E** mRNA expression of Piezo1 in the NG in the ultrasound-stimulated vagus nerve group and sham group. **F** mRNA expression of Piezo2 in the NG in the ultrasound-stimulated vagus nerve group and sham group. (sham: n = 7 rats; aBCI: n = 11 rats). *p < 0.05 by student's t-test. Data are presented as mean ± SEM. **G** Schematic representation of GsMTx4 injected into NG to block MSCs. **H** Representative EEG of saline sham, saline closed-loop aBCI, GsMTx4 sham and GsMTx4 closed-loop aBCI. Seizure detected in real time (green) and sham / closed-loop aBCI stimulation (red). **I** Statistical graphs of average seizure duration for saline sham, saline closed-loop aBCI, and GsMTx4 sham, and GsMTx4 closed-loop aBCI groups. The GsMTx4 MSCs blocker injected to NG disabled aBCI's antiepileptic effects (saline-sham: n = 6 rats; saline-aBCI: n = 8 rats; GsMTx4-sham: n = 7 rats; GsMTx4-aBCI: n = 8 rats). **p < 0.01 by student's t-test. Data are presented as mean ± SEM.

Firstly, we utilized ultrasound with a PRF of 20 Hz and an acoustic pressure of 0.9 MPa in the closed-loop aBCI system. According to the previous study, we found that the duration of a single seizure was about 60 seconds within 30 minutes after PTZ injection, thus we set the ultrasound stimulation duration to 60 seconds (Figure S4A). The results showed that the duration of a single seizure in the sham group was from 21 to 60 seconds; while the duration of a single seizure in the closed-loop aBCI group was from 11 to 40 seconds and was mainly concentrated in the range between 21 and 30 seconds (Figure S4C). Meanwhile, the EEG results showed a 25.7% reduction in duration of a single seizure in the closed-loop aBCI group compared to the sham group. In addition, we found that closed-loop aBCI with acoustic pressure (1.8 MPa) also reduced duration of a single seizure compared to the sham group (Figure S4D). However, there was no significant difference between different acoustic pressures. To ensure safety, we selected acoustic pressure with 0.9 MPa for further study. We then compared the antiepileptic effect of sham stimulation, random aBCI and closed-loop aBCI (Figure 4A). The random stimulation group triggered random ultrasound outputs within 30 minutes after PTZ injected (Figure 4B), while the closed-loop aBCI group triggered ultrasound stimulation of the vagus nerve after seizures were detected. The results showed no significant difference in duration of a single seizure between random aBCI group and sham group, while the closed-loop aBCI group had significantly decreased seizure duration compared to the sham group (Figure 4C, D. Supplementary Movie 1).

We further investigated the mechanism of ultrasound stimulation of vagus nerve in control of epilepsy. Therefore, we hypothesized that ultrasound activates MSCs in NG to inhibit neuronal excitability in brain. Piezos including Piezo1 and Piezo2 are common MSCs within the NG and are involved in regulating physiological activities such as blood pressure 26-28. Therefore, we used RT-qPCR to investigate the role of ultrasound stimulation of the vagus nerve in the regulation of Piezo1 and Piezo2. The results showed that aBCI increased the mRNA expression of Piezo1 and Piezo2, with a statistically significant difference in Piezo2, compared with the sham group (Figure 4E, F). This suggests that MSCs within the NG may be involved in the regulatory effects of aBCI on the vagus nerve.

Then, we injected GsMTx4, an antagonist of MSCs, to block MSCs in the NG, while the control group was injected with saline (Figure 4G). In the saline-treated groups, closed-loop aBCI significantly shortened the duration of a single seizure, whereas in the GsMTx4-administered groups, closed-loop aBCI failed to suppress seizures (Figure 4H, I).

We also found a 27% reduction in duration of a single seizure in the saline-closed-loop aBCI group compared to the GxMTx4-closed-loop aBCI group (Figure 4H, I). These results suggest that blocking MSCs in NG undermines the antiepileptic effect of closed-loop aBCI.

### The aBCI inhibited the abnormal excitability of the limbic system via vagal afferent pathways

**Figure 5:**
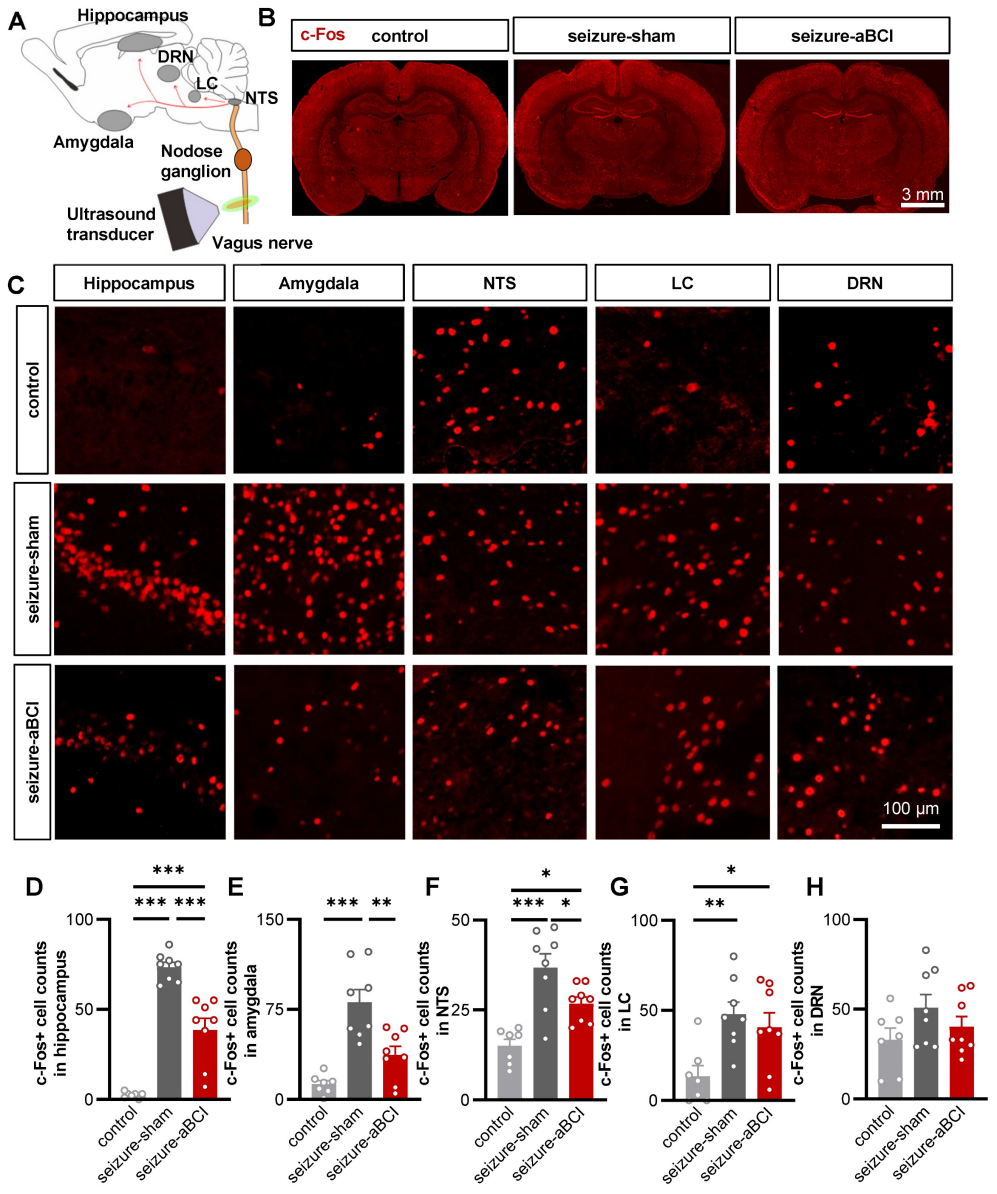
** The aBCI reverses ipsilateral limbic system and vagal pathway abnormal excitability induced by PTZ. A** Diagram showing transmission and modulation pathways in the vagus nerve. **B** Representative c-Fos images in control, sham and aBCI groups. **C** Representative detail maps of nuclei including hippocampus, amygdala, NTS, LC and DRN. The c-Fos protein is marked with red. **D** Statistical graph of c-Fos expression in the hippocampus. PTZ strongly induced abnormal activity in neurons while aBCI reversed the excessive activation. **E** Statistical graph of c-Fos expression in amygdala. The aBCI caused an effect similar to that in hippocampus, inhibiting neuronal hyperexcitability. **F** Statistical graph of c-Fos expression in NTS. **G** Statistical graph of c-Fos expression in LC. **H** Statistical graph of c-Fos expression in the DRN. Number of samples from graph **D** to **H**: control: n = 7 rats; PTZ-sham and PTZ-aBCI: n = 8 rats. *p < 0.05, **p < 0.01, ***p < 0.001 by Tukey's multiple comparisons test following one-way ANOVA. Data are presented as mean ± SEM.

The hippocampus and amygdala are important nuclei of the limbic system and plays a critical role in the onset and development of epilepsy 46, 47. PTZ is a GABA receptor antagonist 48, which causes a decrease in inhibitory synapses and an abnormal increase in neuronal activity until epilepsy develops 49. To further study the central regulatory effect of aBCI, c-Fos expression was observed to evaluate neuronal activities in the limbic system and vagal afferent pathway nuclei. The immediate early gene *c-Fos* is expressed as c-Fos protein when neurons are stimulated, therefore, the degree of neuronal activation can be evaluated by the expression of c-Fos 50. First, we examined the ipsilateral hippocampus and amygdala (Figure 5A-C). For the control group, rats only received the same anesthesia, fixation, and false stimulation, and did not receive PTZ injection to induce seizures. Our results found that the expression of c-Fos was rarely detected in the hippocampus and amygdala of the rats in control group. In stark contrast, we observed robust c-Fos expression in the PTZ-treated group, suggesting that PTZ can induce the neuronal discharges of hippocampus and amygdala. The c-Fos expression in the hippocampus and amygdala in the aBCI group was significantly reduced compared to the sham group (Figure 5D, E). Interestingly, similar trends were found in contralateral hippocampus and amygdala (Figure S5A-D).

Subsequently, we determined whether the vagal afferent pathways, including the nucleus tractus solitarius (NTS), locus coeruleus (LC), and dorsal raphe nucleus (DRN), are involved in the antiepileptic effect of aBCI (Figure 5C). The results showed that PTZ-induced seizures increased c-Fos expression in the bilateral NTS, LC, and DRN, and aBCI stimulation decreased the c-Fos expression in the NTS compared with the sham stimulation (Figure 5F-H; and Figure S5B, E, F). In addition, aBCI showed a trend of downregulating c-Fos expression in the DRN (Figure 5H).

In addition, aBCI cannot inhibit the neuronal discharges after vagotomy (Figure S6), indicating that the intact cervical vagus nerve is essential for aBCI regulation of the vagus nerve pathway.

### The thermal effects of aBCI

To investigate whether the antiepileptic effects of aBCI are related to thermal effects, we measured the temperature of the neck skin changes during aBCI (Figure S7A, B). The 15-minute aBCI marginally increased the local temperature by less than 1 ℃ compared to the sham group (Figure S7C, D). Then, we further investigated the thermal effect of aBCI by implanting a type-k thermometer around the vagus nerve (Figure S7E). It was found that the localized temperature of the vagus nerve increased less than 1 ℃ during aBCI stimulation compared with the sham stimulation group, and the localized temperature of the vagus nerve decreased rapidly after the aBCI stimulation was stopped (Figure S7F).

In addition, to rule out the possibility that thermal stimulation of the vagus nerve can suppress seizures, a metal heater was attached to the skin above the vagus nerve and used to stimulate the vagus nerve (Figure S7G, H). Consistent with our observation, thermal stimulation did not reduce the total number of seizures and total seizure duration (Figure S7I, J). These results indicated that thermal effects were not the major contributor to seizure suppression by aBCI.

### Safety assessment of aBCI

The MRI and H&E staining were used to evaluate the safety of aBCI. The MRI results showed that the brain structure of the rats was intact after ultrasound stimulation, with no signs of edema or necrosis (Figure S8A). According to the H&E staining results, the hierarchical structure of the vascular wall was clear, the endothelial cells of the blood vessels were arranged neatly without breakage, the nerve fibers of the vagus nerve were arranged intact, the nuclei were clear, and there was no sign of inflammation or necrosis (Figure S8B). Therefore, aBCI is a safe neuromodulation technology.

## Discussion

In this study, we developed a prototype of closed-loop aBCI system to inhibit PTZ-induced seizures in rats. We demonstrate for the first time that ultrasound selectively actives mechanosensitive neurons in the NG and suppress abnormal neuronal excitability in the hippocampus and amygdala via the vagus nerve pathway. The development of closed-loop aBCI provide a non-invasive treatment route for patients with epilepsy.

Compared to open-loop stimulation, closed-loop aBCI adjusts ultrasound stimulation based on seizure status 51. In this study, ultrasound will be triggered once detecting a seizure, and EEG recorder continue to dynamically monitor the epileptic discharges. If epileptic discharges were still detected, ultrasound stimulation signals will be sequentially output. The output will stop once the epileptic discharges were inhibited.

In the neural system, the immediate-early gene *c-Fos* is expressed as the c-Fos protein when neurons are activated 18, 50. In animal models of epilepsy, c-Fos expression of NTS is elevated due to inflammation 52 or neural activities 53. As reported by Kang et al. seizures violently activate nuclei throughout the brain 37. The c-Fos staining was used to investigate the different effects on vagal pathway between acute VNS and aBCI (Figure S9A). Compared to the sham group, there was no significant difference on either side of the amygdala and hippocampus (Figure S9B, C), and aBCI further reduces the c-Fos expression in NTS, LC and DRN bilaterally. However, VNS upregulated c-Fos expression in the bilateral the NTS, LC, and DRN compared to the sham group (Figure S9D-F). In terms of the results, aBCI may control seizures by inhibiting the abnormal activation of the vagal loop and thereby controlling epileptic seizures. This suggests that the regulatory effects and mechanisms on the vagus nerve were different between aBCI and VNS.

Previous studies have found that the efficacy of VNS treatment is closely dependent on the duration of treatment, with short periods of VNS treatment showing limited effect to suppress seizures 54, 55. However, over 39% epilepsy patients receiving VNS had achieved a seizure control rate of 50% after 3-month-long regular stimulation 8, 56. A possible explanation is that ultrasound downregulated a broader range of neurons, including neurons in the NTS, LC, DRN, amygdala, and hippocampus of seizure rats, while VNS upregulated neuronal activity in nuclei such as the NTS and LC, and downregulated neuronal activity in the hippocampus (Figure S9). Therefore, a short period of VNS is insufficient to control PTZ-induced acute seizures.

Previous studies have shown that ultrasound modulates neuronal activity by MSCs 57, 58. Our study found that ultrasound stimulation of the vagus nerve upregulated the mRNA expression of Piezo1 and Piezo2 within the NG, especially the mRNA expression of Piezo2 was significantly increased, however, injection of GsMTx4 in the NG abolished the antiepileptic effect of closed-loop aBCI (Figure 4E-I). Previous studies have been demonstrated that vagus nerve sensory neurons are located in the NG 25, which contains of chemosensitive and mechanosensitive neurons 59, 60. The mechanosensitive neurons in the NG express MSCs such as Piezo1 and Piezo2 61, 62. Recently, a study demonstrated that Piezo2 was essential for ultrasound stimulation to evoke firing in a subpopulation of sensory neurons in mice, and that this ion channel overall decreased the threshold of sensory neurons responding to ultrasound stimulation 30. The present study suggests that MSCs, especially Piezo2, play a crucial role in the antiepileptic effects of ultrasound stimulation of the vagus nerve.

The NTS acts as a gateway to the brain for vagal afferents, receiving vagal sensory information afferents which are then relayed to other brain regions via ascending neural projections to modulate brain function 63, 64. Although the mechanism of VNS to control epilepsy remains unclear, it is generally accepted that increased release of norepinephrine via the NTS-LC pathway is an important mechanism 65. Vagal signaling is transmitted to the LC followed by projections to the DRN, which then projects to the limbic nuclei and modulates hippocampal and amygdala activity 66-68. In our study, we found that acute aBCI extensively inhibited seizure-induced abnormal excitability in brain regions associated with vagal pathway, such as the NTS, LC and DRN (Figure 5). Further studies are needed to investigate how aBCI modulates NTS activity and projects to other nuclei to exert antiepileptic effects.

Designing a non-invasive BCI is a central task in the comprehensive analysis and understanding of the brain, which is one of the most important challenges in neuroscience. Currently implanted BCI are cranial and invasive and have become a serious bottleneck in applications. The development of new non-invasive reading and writing technologies will advance significant innovations and breakthroughs in the field of BCI. Besides EEG recordings, we further studied functional ultrasound brain imaging to non-invasively "read" neural activity in the brain, and apply acoustic radiation force to precisely "write" neural information, realizing a non-invasive closed-loop aBCI. The implementation of non-invasive closed-loop aBCI is expected to provide new tools in the fields of bioacoustics, life health and rehabilitation, brain science, brain diseases, driverless, and artificial intelligence.

## Conclusion

In summary, we developed a noninvasive closed-loop aBCI that noninvasively and specifically stimulates the vagus nerve for seizure control. We demonstrated that closed-loop aBCI can "read" the brain activity by EEG and apply acoustic radiation force to stimulate vagus nerve to precisely "write" neural information, realizing a non-invasive closed-loop aBCI. In addition, our results show a practical demonstrate a closed-loop aBCI with potential applicability to epilepsy. We anticipate that non-invasive closed-loop aBCI can be used for other brain disorders and provide a powerful therapeutic and neural-interface technique.

## Methods

### Animal preparation

All experimental protocols were approved by the Animal Experiment Ethics Committee of the Shenzhen Institute of Advanced Technology, Chinese Academy of Sciences (certificate number: SIAT-IRB-150213-YGS-ZHR-A0094-2). The male Sprague-Dawley rats (200-220 g, Beijing Vital River Laboratory Animal Technology Co., Ltd.) were used in the experiment. Rats were housed in a temperature-controlled room (25 ± 1 ℃) with a 12-hour light / dark cycle and free access to food and water. Every effort was made to minimize animal use and pain throughout the study.

### Vagus nerve localization by imaging

A 9.4 T MRI scanner (United Imaging, uMR 930) with a 72 mm inner diameter volume coil for radiofrequency transmission and a single-channel array head coil for signal reception. Anesthesia was induced using isoflurane (RWD Life Science, 3 %, 1 L/min) and maintained using isoflurane (1.5 %, 1 L/min). Body temperature was maintained using a heated waterbed. T2-weighted high-resolution multilayer fast spin echo (FSE) sequences were used for cervical vascular and neuroimaging with the following sequence parameters: TR = 550 ms, TE = 39.52 ms, ETL = 15, BW = 300 Hz/pixel, field of view: 30 × 35 mm^2^, matrix size 412 × 480, slice thickness = 0.5 mm, slice gap = 0 mm. The common carotid artery and vagus nerve were then further differentiated by T1-gre-quick 3D. Sequence parameters were set as follows: TR = 15.0 ms, TE = 2.35 ms, BW = 250 Hz/pixel, field of view: 36 × 35 mm^2^, matrix size 560 × 544, slice thickness = 0.2 mm. On the other hand, a high-frequency ultrasound system (VisualSonics, Vevo 2100) with 40 MHz linear array probe (VisualSonics, MS550D) was used to localize the vagus nerve again.

### Vagus nerve resection

The rats were anesthetized with isoflurane (2 %, 1 L/min) and fixed in the supine position on a rat bench. The neck hair was removed and the skin was disinfected. Next, the skin was incised and the muscles were separated. Then, the left vagus nerve was located behind the sternocleidomastoid and carefully separated from the common carotid artery before being cut off a section. Finally, the skin was closed with suture material. Rats were used for the latter experiment after recovering for 10 days.

### RNA extraction

Vagus ganglia were taken into 1.5 ml centrifuge tubes and 1 ml of RNA extraction reagent (Aikairui Biotechnology, AG21102) was added to each tube, which was subsequently ground in a cryomill and the above mixture was transferred to centrifuge tubes and left to stand for 5 min at room temperature. The mixture was then centrifuged for 5 min (12,000 rpm, 4 ℃), the supernatant was aspirated and transferred to a new centrifuge tube, chloroform was added and mixed thoroughly, and the mixture was left to stand for 5 min at room temperature, then centrifuged for 15 min (12,000 rpm, 4 ℃) and the supernatant was extracted. Add isopropanol, mix thoroughly, allow to stand for 10 minutes at room temperature, then centrifuge for 10 minutes (12,000 rpm, 4 ℃) and discard the supernatant. Add 80% ethanol (-20 ℃) to the precipitate and centrifuge for 5 minutes (7,500 g, 4 ℃) to discard the supernatant. The supernatant was discarded and dried at room temperature for 5 min before adding an appropriate amount of RNase free water to the centrifuge tubes to dissolve the RNA. Nanodrop one).

### RT-qPCR detection

Firstly, there was RNA was performed to remove gDNA by adding gDNA clean reaction Mix mix (Aikairui Biotechnology, AG11728) (42 ℃, 2 min). Then 5X Evo M-MLVRT Reaction Mix (Aikairui Biotechnology, AG11728) was added and placed in a PCR instrument (Thermo Fisher Scientific, MiniAmp Plus) for reverse transcription (37 ℃, 15 min; 85 ℃, 5 sec). The RNA was then transcribed into cDNA, and the primers for the target genes (*Piezo1* and *Piezo2*) and the internal reference gene β-actin were added and used to generate a melting curve using RT-qPCR (95 ℃, 30 sec, 1 cycle; 95 ℃, 5 sec, 40 cycles; 60 ℃, 30 sec, 3 cycles) to amplify and quantify the cDNA of the target genes.

The primer sequences were as follows: *Piezo1*: CGGACAGTGAGGAGGAAGAGGAG (5' to 3'), CCTGTTCACGACGCTGCCTT (3' to 5'); *Piezo2*: AGCCTCCCAGACCTAGCCATTATG (5' to 3'), TCTTTCCCAGCCTCACCCTTCTC (3' to 5'); β-actin: CCCATCTATGAGGGTTACGC (5' to 3'), TTTAATGTCACGCACGATTTC (3' to 5').

### Chemical blocking the MSCs channel in NG

Rats were anesthetized with ketamine and fixed as described above. The rat's neck hair was removed and the skin was disinfected by iodophor. Imaging ultrasound was used to localize the vagus nerve as described above. The nodose ganglion was located at the exit of the jugular foramen. The GsMTx4 (abcam, ab141871, 100 μmol/L, 50 ul) was injected around the NG and used to block the MSCs. The control group was injected with an equal amount of saline. The subsequent study was performed 5 minutes after the GsMTx4 or saline injection.

### Electroencephalogram electrode implantation

The rats were anesthetized with isoflurane, fixed using stereotaxic apparatus. The stainless-steel micro-nail electrodes for EEG recording were positioned in the skull above the hippocampus, the reference electrode was anchored in the skull above the prefrontal cortex, and the ground electrode was placed on the skull over the cerebellum. Finally, dental cement was applied to fix the electrode. The rats were given food and water after the operation, and follow-up experiments were conducted after 1-3 days of recovery.

### EEG recording and analysis

An EEG recording system (Solar, 1848) with a sampling rate of 500 Hz was used to record EEG of rat. A wireless EEG recorder with a sampling rate of 125 Hz was used for the closed-loop aBCI system. The bandpass filter for data acquisition was set between 0.01 and 70 Hz. Including the number of seizures and seizure duration were used to assess the antiepileptic effect of closed-loop aBCI.

### Acute seizure model establishment

A 10-minute EEG was recorded as a baseline, and then the rats were anesthetized by intraperitoneal injection of ketamine (Beikang Pharmaceutical Industry, 50 mg/kg) and fixed as described above. Acute seizures were induced by of PTZ (Sigma, P6500, 100 mg/kg, intraperitoneal injection). Rats would develop Racine IV or V grade seizure within 2 hours. In the first 30 min, rats were fixed on the brain stereotactic apparatus preventing behavioral observations, thus we determine the number and duration of seizures according to the EEG signals.

### Establishment of a closed-loop aBCI system

We establish closed-loop aBCI systems to provides support for online EEG decoding analysis. Our software runs on a desktop PC (Intel Xeon E5-1620 CPU, 3.5 GHz, 32 GB RAM, Window OS). The components and tasks are managed using the aBCI system client software and include the following modules: 1) wireless EEG recorder, 2) serial data acquisition and cache, 3) brain signal decoder, 4) ultrasound device controller, and 5) acoustic vagus nerve stimulator. The software was developed using the C# programming language. The encoder contains a multi-level threshold model, an LSTM model, and an SVM model, which are implemented as follows.

### Multi-level threshold algorithm model

Before closed-loop aBCI experiments, we obtained 3000 samples of EEG signal data from rats collected within 30 minutes after PTZ injection, each with a duration of 3 seconds, including 2869 non-seizure samples and 131 seizure samples. Then, 6 features including instantaneous frequency mean, spectral entropy mean, Euclidean norm, coastline (CL), standard deviation (STD), and logarithm of energy entropy (E_log_) were extracted for each sample both seizures and non-seizures. In addition, we normalized the feature set using the normalize function in MATLAB, which scales the matrix to the range [0,1]. Based on the statistical results, we selected CL, STD, and E_log_ features, and then designed a multilevel thresholding model based on these three features.

Offline, we split the seizure and non-seizure data from the first 30 min of each rat into 600 data samples according to a 3 s time window, and labeled it as either seizure or non-seizure. A total of 4200 samples were obtained, of which 100 were seizures and 4100 were non-seizures samples. From these, 100 seizure samples and 100 non-seizure samples were taken. We divided the total 200 datasets into 160 training sets and 40 testing sets in the ratio of 8:2 using the dividerand function (MATLAB). We then used the feature minimum of the 80 epilepsy samples in the training set as the first stage threshold corresponding to the features, and the mean as the second stage threshold corresponding to the features, and then applied the thresholds to the multilevel thresholding model. Our definition is that the multilevel thresholding model judges a seizure when all three features are greater than their corresponding first stage thresholds, or when two of them are greater than their corresponding second stage thresholds, and otherwise judges a non-seizure. Finally, we input 40 test sets into the multilevel threshold model for judgement and get the accuracy.

Online, the acquired EEG signals are passed through a third-order IIR filter, and then the values of CL, STD and E_log_ were calculated in parallel. Given a sequence of EEG signals x(i), i = 1,...,N, its calculation formula is as follows:



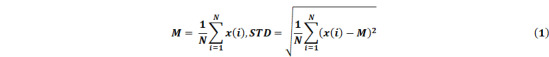





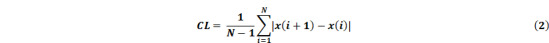





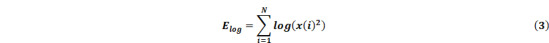



All features are computed every 3s, i.e. the length of the detection window n = 375. Each feature value is then compared with the corresponding threshold for seizure detection. When all three features in the channel are greater than the first stage threshold or two of them are greater than the second stage threshold, the multilevel threshold model considers that there is an epileptic seizure in the channel, and will trigger the ultrasound stimulation.

### Long short-term memory (LSTM)

We apply EEG data to LSTM for classification. The network contains three inputs. X_t_ is the current time step of the input, H_t-1_ is the output of the previous LSTM cell, and C_t-1_ represents the memory of the previous cell. Similarly, H_t_ and C_t_ are the output and memory of the current block, respectively. We tested the LSTM for the optimal number of memory cells to obtain the highest accuracy. In the LSTM architecture, we have two main hyperparameters that can influence the accuracy of epilepsy detection: the number of memory blocks and LSTM layers. The Adam optimization in the activation layer has been widely regarded as the best choice since its inception. A good learning rate for Adam optimization is either 0.01 or 0.001. In our case, a learning rate of 0.001, a sequenceInputLayer of 1, an lstmLayer of 250, and a fullyConnectedLayer of 2 provide better accuracy. We split the seizure and non-seizure data from the first 30 min of each rat into 600 data samples according to a 3 s time window, and labeled it as either seizure or non-seizure. A total of 4200 samples were obtained, of which 100 were seizures and 4100 were non-seizures samples. From these, 100 seizure samples and 100 non-seizure samples were taken. We divided the total 200 datasets into 160 training sets and 40 testing sets in the ratio of 8:2 using the dividerand function (MATLAB). We run 160 training sets into the trainNetwork function for training and 40 testing sets put into the classify function for testing.

### Support vector machine (SVM)

SVM is a statistically generated machine learning method that has good classification and prediction capabilities. In this work, we apply the same EEG datasets as multilevel thresholding and LSTM to SVM for classification. Firstly, we split the seizure and non-seizure data from the first 30 min of each rat into 600 data samples according to a 3 s time window and labeled it as either seizure or non-seizure. A total of 4200 samples were obtained, of which 100 were seizures and 4100 were non-seizures samples. From these, 100 seizure samples and 100 non-seizure samples were taken. We divided the total 200 datasets into 160 training sets and 40 testing sets in the ratio of 8:2 using the dividerand function (MATLAB). Next, we compute the mean instantaneous frequency, the mean spectral entropy, the Euclidean norm, the coastline, the standard deviation, and the logarithm of the energy entropy for each sample of raw EEG signals. Then, we normalized the feature set using the normalize function which scales the matrix to be in the range [0,1] in MATLAB. Finally, the parameters of the kernel function use the rbf function, and we put 160 training sets into the fitcsvm function, which automatically optimum the hyperparameters to train the model, then we put 40 testing sets into the predict function to call the model to get the classification accuracy.

### Comparison of three decoder strategies

In order to better evaluate the classification results, this paper adopts a confusion matrix evaluation method and uses three criteria, sensitivity (Sen), specificity (Spe) and accuracy (Acc), as evaluation indicators, calculated as follows:



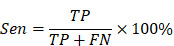





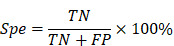





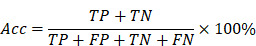



where true positive (TP) is the number of seizure epochs determined by both algorithm and doctor. True negative (TN) is the number of non-seizure epochs determined by both algorithm and doctor. False negative (FN) is the number of seizure epochs misclassified by algorithm, i.e. recognized as non-seizure but actually they are seizures. False positive (FP) is the number of non-seizure epochs misclassified by algorithm, i.e. recognized as seizure but actually they are non-seizures.

### Controller for triggering ultrasound output

The Arduino acts as a controller for the ultrasound generator, which is a triggered ultrasound output that is achieved by switching between high and low levels on specific pins. When the seizure was detected, a trigger signal was sent to the ultrasound signal controller. During the experiments, sham group was not stimulated, closed-loop aBCI was ultrasonically stimulated according to the epilepsy detection, and random aBCI matched the same sequence of parameters as the closed-loop aBCI. For random aBCI, we generated a random sequence by calling a random function every 3 seconds with a polling clock, presetting one sequence as the trigger stimulus condition and none of the other sequences.

### Ultrasound stimulation

In this study, we used a 250 kHz focused ultrasound transducer (Sonic Concepts, H-115). A signal generator (RIGOL, DG4062) and a power amplifier (E&I, 2100 L) set the stimulation parameters and drive the transducer. The focal width and focal length of the H-115 transducer were 4.5 and 39.5 mm (-6 dB), respectively. A coupling cone filled with degassed water was used to couple the transducer to the skin. The ultrasonic parameters were as follows: pulse-repetition frequencies (PRF) = 1 / 20 / 1000 Hz, duty cycle (DC) = 10%, sonication duration (SD) = 5 s, inter-stimulus interval (ISI) = 5 s, insonation time = 15 / 30 minutes, acoustics pressure (AP) = 0.45 / 0.90 / 1.80 MPa, and spatial peak temporal average intensity (I_spta_) = 82.2 / 328.7 / 1314.9 mW/cm^2^. The different combinations of ultrasonic parameters we used are listed in Table 1.

### Electrical stimulation of vagus nerve

To investigate the regulation of PTZ-induced acute seizures by VNS and closed-loop aBCI, we surgically implanted vagus nerve electrodes and performed experiments after 10-day-long recovery (Figure S3A-C). The vagus nerve was fixed in a cuff electrode (Pins medical, LR07) consisting of a bipolar platinum / iridium needle, and the cuff electrode connector was placed under the skin. The bipolar stimulation sleeve electrode uses the cathode as the proximal lead and the anode as the distal lead. The animals took approximately 10 days to recover after the surgery. The acute-seizure rats were fixed to the fixator, as described previously. The vagus nerve stimulation electrode was connected to an external controller (Pins medical, LR07). After 15 minutes of PTZ injection, the left vagus nerve was continuously stimulated for 15 minutes (one burst of 20 Hz with 250 ms, pulse-width of 2.0 mA output current).

### Specimen collection

All rats were deeply anesthetized with isoflurane and fixed in the supine position on the dissection table after the experiment. After opening the thoracic cavity to expose the heart, the right atrium was incised. Rats were sequentially perfused with phosphate buffer saline (PBS) and paraformaldehyde through the left ventricle. After completing perfusion, the extracted brains were fixed in 4% paraformaldehyde and immersed for 24 hours. Brains were dehydrated in gradient sucrose solutions (20%, 40%).

### Immunofluorescent staining

The c-Fos expression was used to investigate the effect of aBCI on the vagus nerve pathway and limbic system. The brains were cut on a cryostat into 30 µm coronal frozen sections using a freezing microtome (Leica, CM1950) and stored at -20 ℃. And then, the brain sections were washed in PBS 3 times for 10 minutes each, blocked with 5% bovine serum albumin (BSA, Amresco, A7030) and 0.5% triton (Sigma, T8787) for 2 hours at 4 ℃. Then primary antibody c-Fos (synaptic systems, 226003, 1:1000) was added and incubated overnight, then secondary antibody (Beyotime Biotechnology, A0453, 1: 1000) was added and incubated for 3 hours. Then, the nuclei were stained with DAB (Beyotime Biotechnology, C1006). After the immunofluorescence histochemical staining, the sections were observed and images were captured using VS200 microscope (Olympus, VS200). The c-Fos positive cells were counted in the brain regions of interest, including bilateral hippocampus, amygdala, NTS, LC and DRN.

### The thermal effects assessment

We also assessed the impacts of thermal effects during aBCI by a thermal infrared imager (NEC Avio, R300). Temperature measurements were logged every minute during ultrasound stimulation. Temperature variations between before and after ultrasound stimulation were used to perform the statistical analysis.

To study the heat of aBCI localized in the vagus nerve, we implanted a temperature detector of a type-k thermometer (TES Electrical Electronic, 1310) around the left vagus nerve of rats. Then, the rats were anesthetized with isoflurane (1.5%, 1 L/min) and the vagus nerve temperature was recorded every minute for 15 minutes as a baseline, followed by a 15-minute aBCI. Finally, the temperature of the vagus nerve was recorded for 15 minutes after aBCI. We used the temperature data collected 15 minutes before aBCI as a baseline to study the localized temperature changes in the vagus nerve during aBCI.

Next, a semiconductor heater was placed on the neck of the rat and was used to stimulate the vagus nerve for 15 min with a constant temperature. Antiepileptic effect of thermal stimulation of the vagus nerve assessed by comparing the number of seizure and seizure duration in the thermal stimulate group with those in the sham group.

### Safety assessment

Imaging and tissue staining for investigating the safety of ultrasound stimulation of vagus nerve. For imaging, rats were scanned on a 9.4T MR system to assess the safety of brain. A circular, 20 mm receive-only surface coil was placed over the head. The animals were then situated with their heads near the isocenter of the magnet within an 84 mm quadrature transmit receive volume coil. T2-weighted high resolution multi-slice fast spin echo (FSE) sequence was used. Sequence parameters were set as follows, TR = 5402 ms, TE = 26.96 ms, ETL=17, BW = 200 Hz / pixel, field of view: 39 × 40 mm^2^, matrix size 696 × 736, slice thickness = 0.3 mm, slice gap = 0.1 mm.

For tissue staining, a total of 6 rat samples were used for safety evaluation (3 in the sham group and 3 in the aBCI group). Firstly, the common carotid artery and vagus nerve tissues were prepared into 5 μM paraffin sections were stained using a standard H&E staining scheme.

### Statistical analysis

Data were statistically analyzed using GraphPad Prism (version 8.0) and variables were expressed as the mean ± SEM. The Shapiro-Wilk test was used for normality testing. If the data were normally distributed, one-way analysis of variance (one-way ANOVA) and the student's t-test were used to analyze the significance of differences, respectively. Mann-Whitney U and Kruskal-Wallis test were used for variables with non-normal distributions. Statistical significance was set at *p* < 0.05.

## Supplementary Material

Supplementary figures.

Supplementary movie.

## Figures and Tables

**Table 1:** Ultrasonic stimulation parameters

No.	Fundamental frequency (kHz)	PRF (Hz)	DC (%)	SD (s)	ISI (s)	Insonation time (min)	Acoustic pressure (MPa)	I_spta_ (mW/cm^2^)
1	250	20	10	5	5	1	0.9	328.7
2	250	20	10	5	5	1	1.8	1314.9
3	250	20	10	5	5	15	0.9	328.7
4	250	20	10	5	5	15	0.45	82.2
5	250	20	10	5	5	30	0.9	328.7
6	250	1	10	5	5	15	0.9	328.7
7	250	1000	10	5	5	15	0.9	328.7

PRF: pulse repetition frequency; DC: duty cycle; SD: sonication duration; ISI: inter-stimulus interval; spatial peak temporal average intensity (I_spta_).
