# Supplementary material for: Noninvasive closed-loop acoustic brain-computer interface for seizure control
Source: Theranostics. 2024 Sep 9;14(15):5965–81. doi: 10.7150/thno.99820 (PMC11426232; doi:10.7150/thno.99820)
Supplement: Supplementary file 1 — Supplementary figures. [file thnov14p5965s1.pdf]

# Supplementary Material

## Noninvasive closed-loop acoustic brain-computer interface for seizure control

*Junjie Zou*[#1,2], *Houminji Chen*[#1,2], *Xiaoyan Chen*[#2,3], *Zhengrong Lin*[2,3], *Qihang Yang*[2], *Changjun Tie*[2], *Hong Wang*[4], *Lili Niu*[2,*], *Yanwu Guo*[1,*], *Hairong Zheng*[2,*]

*Corresponding author. Email: ll.niu@siat.ac.cn, eguoyanwu@163.com, and hr.zheng@siat.ac.cn

**This file includes:**

Figures. S1 to S9

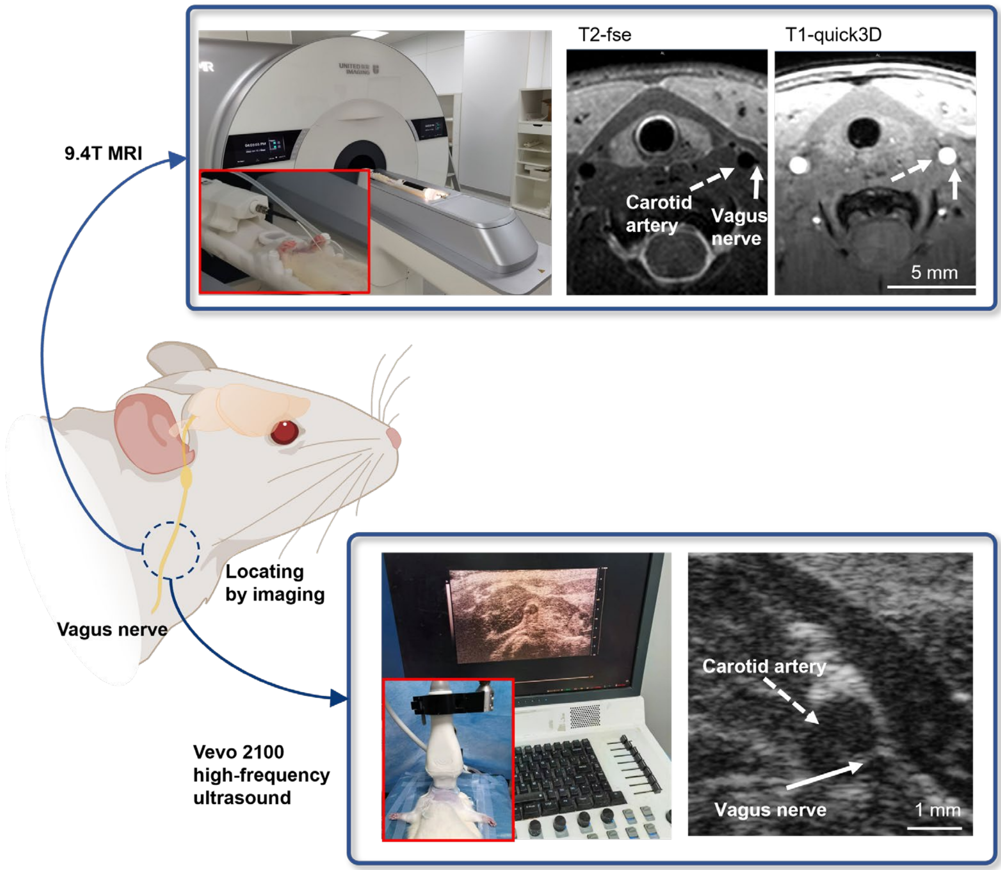


**Figure S1. Localization of the vagus nerve.**

Localization of the cervical vagus nerve using a 9.4T MRI system and a high frequency ultrasound system. The cervical vagus nerve (solid arrow) was located based on T2 weighted fast spin echo sequence. Due to the presence of changes in blood flow within the vessels, the T1-quick 3d sequence of 9.4T MRI was used to differentiate the vagus nerve from the neck vessels. In addition, high-frequency ultrasound was used to observe the activity of the carotid arteries (dashed arrow) in real time, which further determined the location of the vagus nerve.

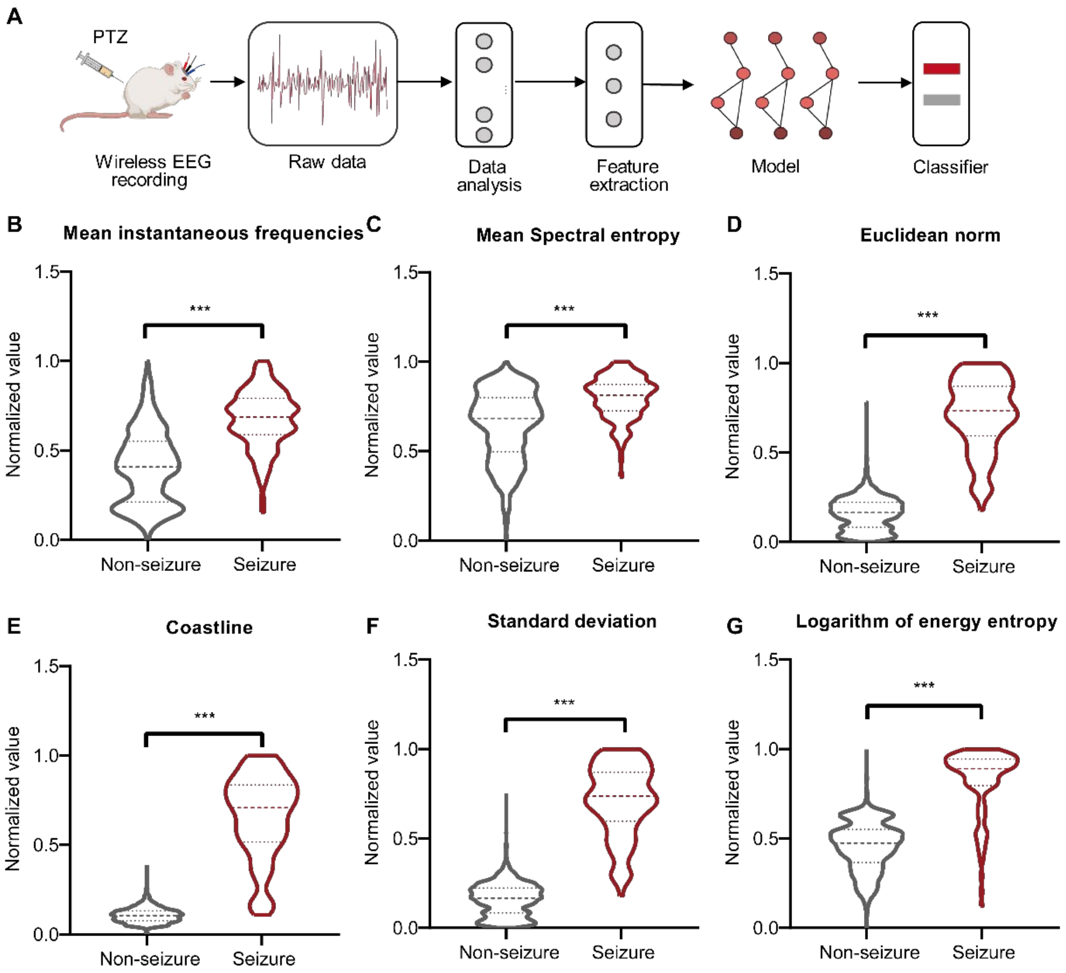


**Figure S2. Features of PTZ induced seizure EEG.**

**A** Flowchart of EEG signal processing. **B** to **G** Statistical graph of mean instantaneous frequencies, mean spectral entropy, Euclidean norm, coastline, standard deviation and logarithm of energy entropy between non-seizure and seizure groups. Non-seizure groups (n = 2869) and seizure (n = 131). Non-seizure versus seizure, $***p < 0.001$. Mann-Whitney U-test was used as significant test.

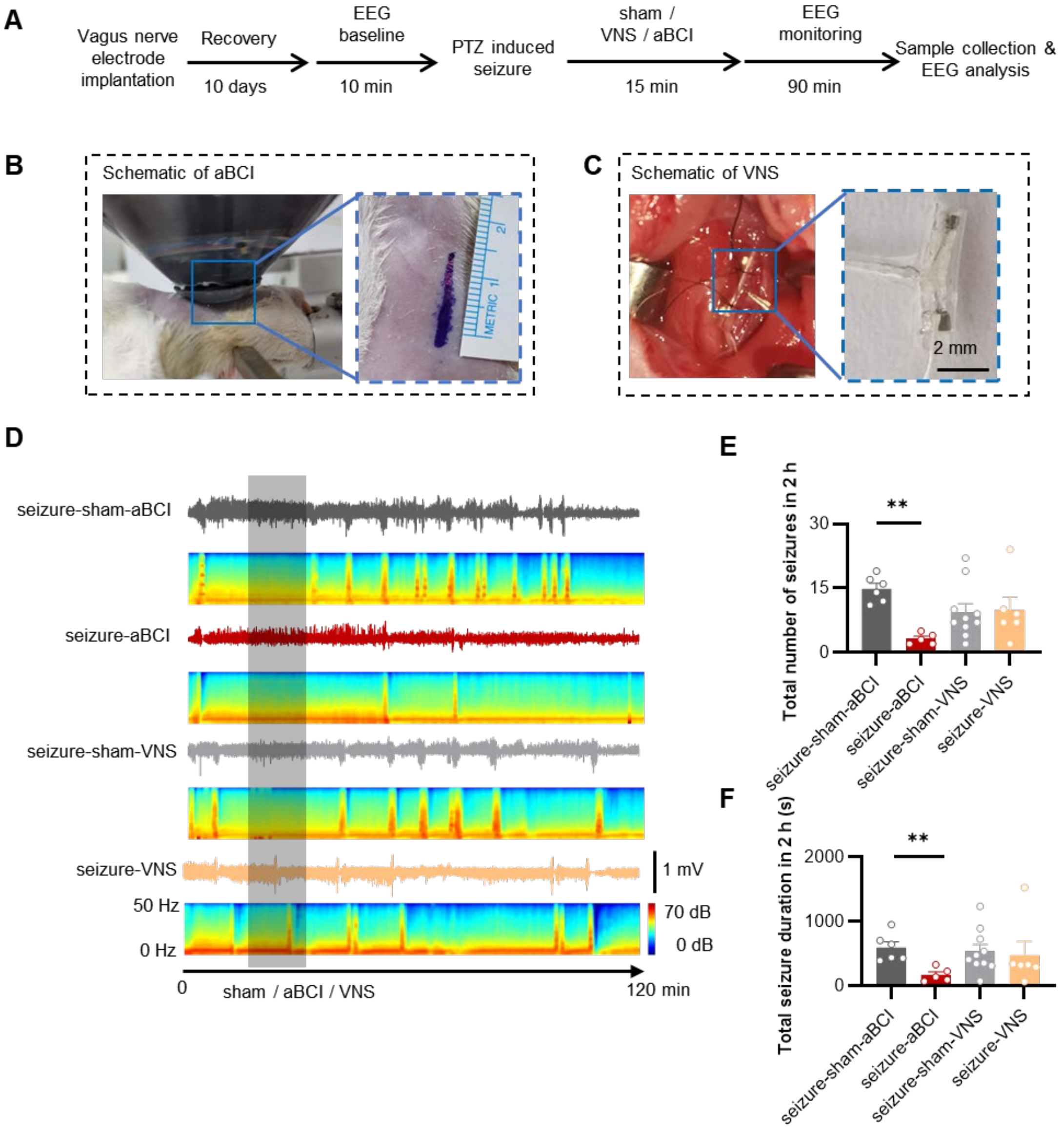


**Figure S3. Antiepileptic effects between aBCI and VNS.**

**A** Schematic of VNS and aBCI experiments. **B** Schematic of aBCI non-invasive stimulation of the vagus nerve. **C** Schematic of the vagal nerve electrode implantation procedure. **D** Representative EEG. The sham-aBCI, aBCI, sham-VNS or VNS 15 min (shadow). **E** Statistical graphs of total number of seizures in 2 hours. aBCI reduced the number of seizures while VNS had a relatively limited effect. **F** Statistical graphs of total seizure duration in 2 hours. VNS had the trend to control the total seizure duration. Sham-aBCI: n = 6 rats, aBCI: n = 5 rats, sham-VNS: n = 10 rats, VNS: n = 6 rats. ***p* < 0.01, Mann-Whitney U-test. Data are presented as mean ± SEM.

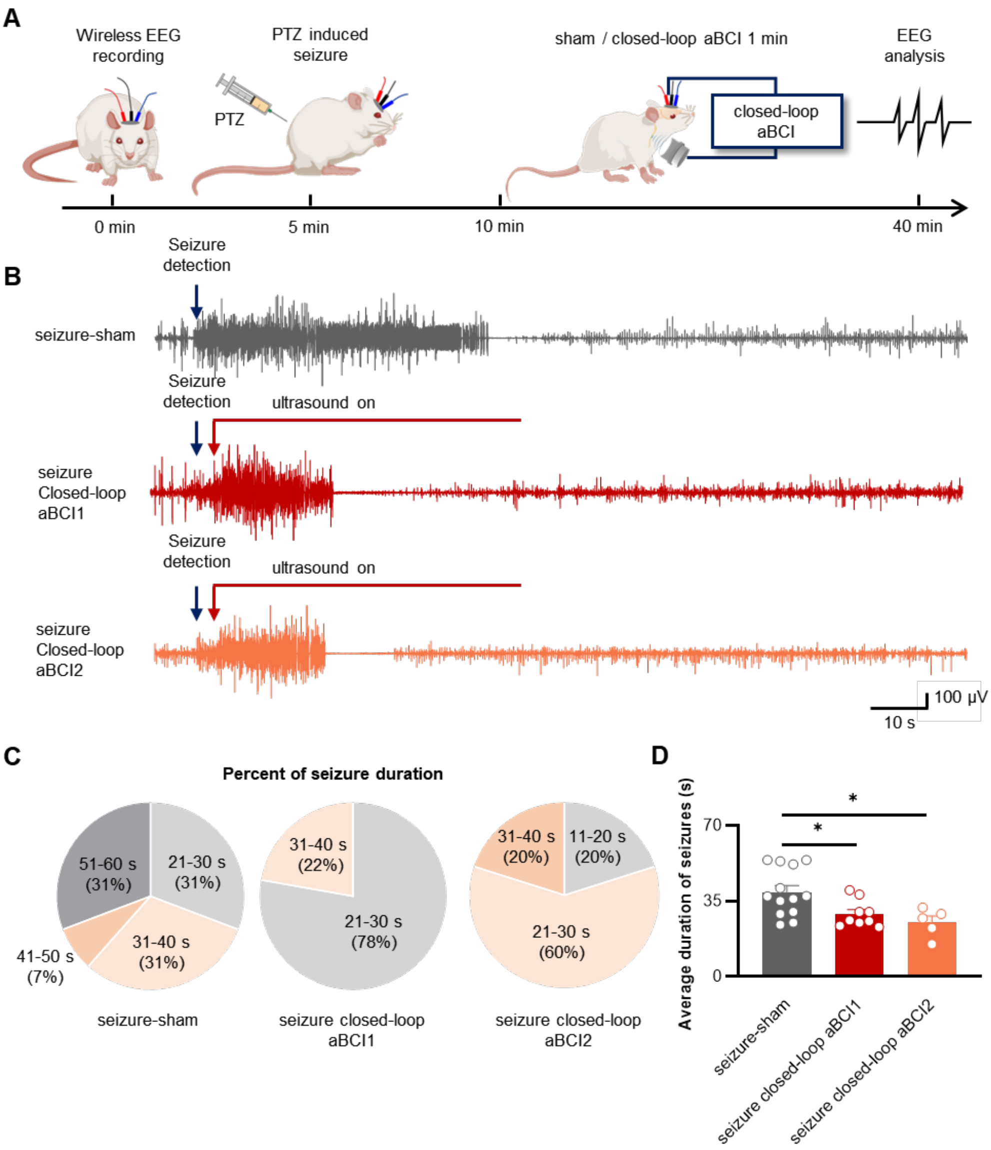


**Figure S4. Seizure inhibition by closed-loop aBCI with difference acoustic pressure.**

**A** The experimental procedures. **B** Representative EEG. Seizure detected (blue) and ultrasound output (red); aBCI 1: closed-loop aBCI (0.9 MPa); aBCI 2: closed-loop aBCI (1.8 MPa). **C** Percent seizure duration. **D** The closed-loop aBCI, both under the two intensities, could reduce seizure duration. There was without significant between two closed-loop aBCI groups. Sham: n = 13 rats; aBCI 1: n = 9 rats; aBCI 2: n = 5 rats. *$p < 0.05$ by Tukey's multiple comparisons test following one-way ANOVA. Data are presented as mean ± SEM.

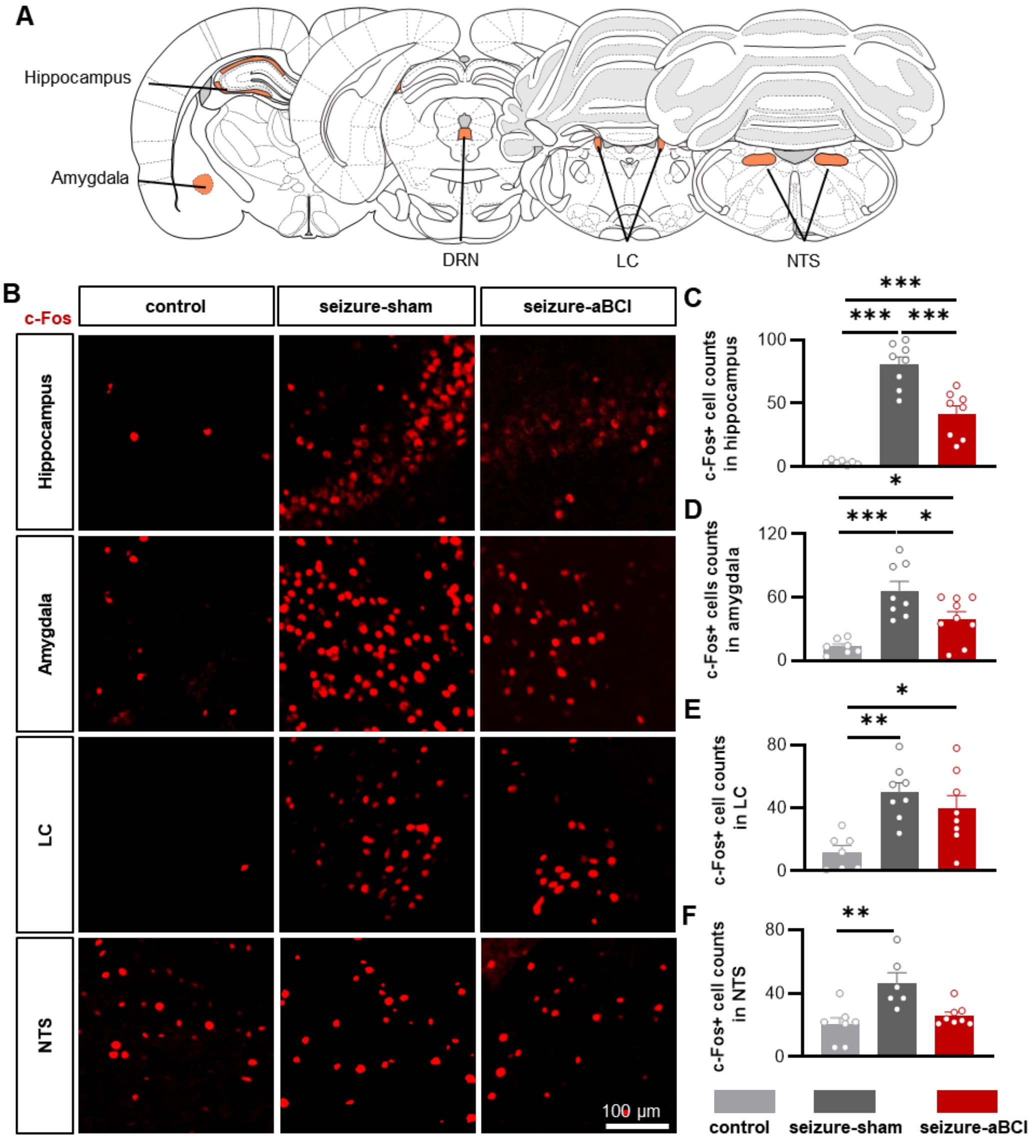


**Figure S5. Regulation of the contralateral brain nucleus by aBCI.**

**A** Schematic representation of brain functional areas related to the vagus nerve. **B** Representative c-Fos immunofluorescence image. **C** to **F** Statistical graph of c-Fos expression in the hippocampus, amygdala, LC and NTS. aBCI reversed PTZ-induced neuronal hyperexcitability in multiple nuclei. Number of samples from graph **C** to **F**: control: n = 7 rats; PTZ-sham: n = 8 rats; PTZ-aBCI: n = 8 rats. *$p < 0.05$, **$p < 0.01$, ***$p < 0.001$ by Tukey's multiple comparisons test following one-way ANOVA. Data are presented as mean ± SEM.

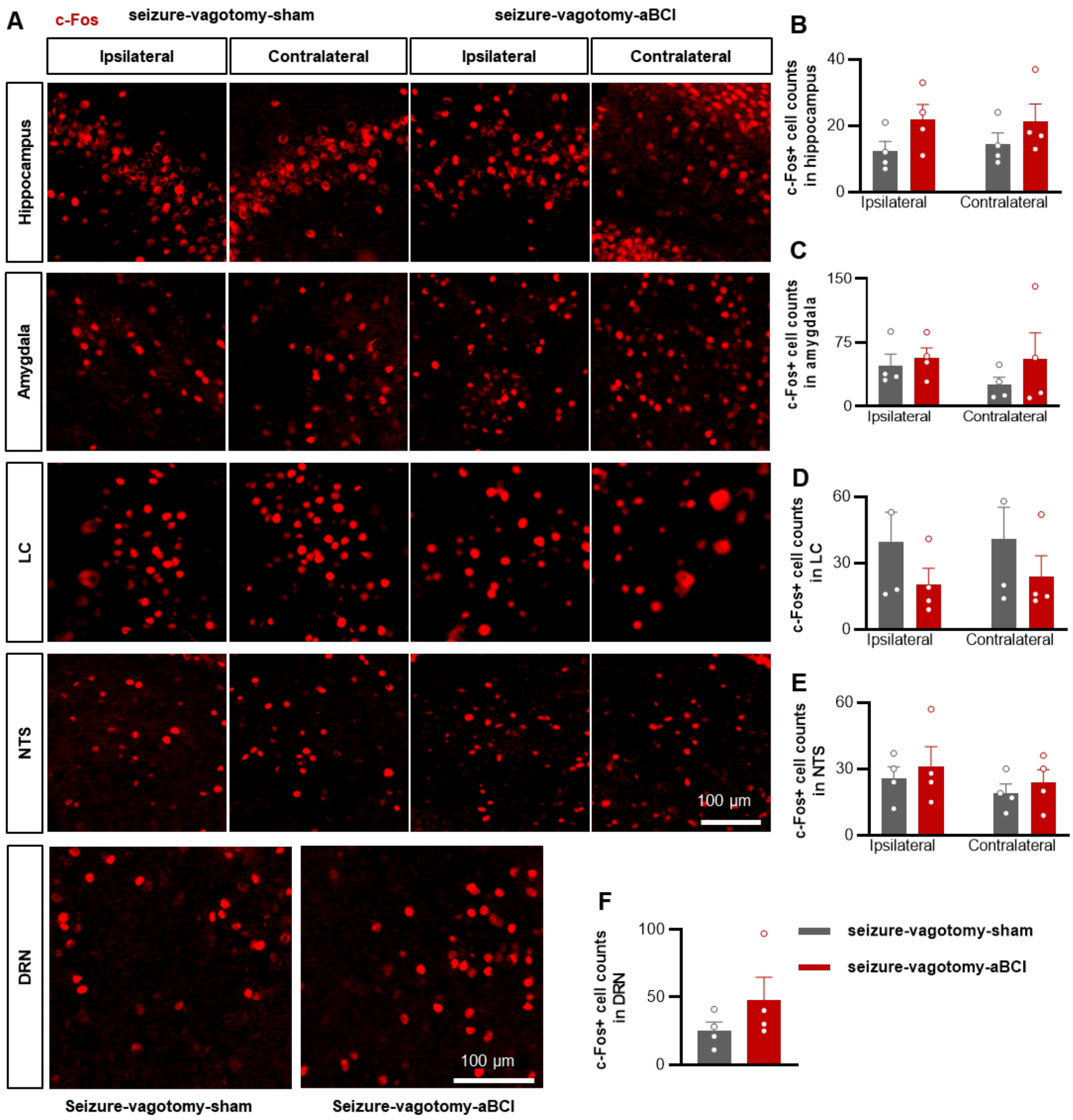


**Figure S6. Regulation of the brain nucleus by aBCI with vagotomy.**

**A** Representative c-Fos immunofluorescence image. **B** to **E** Statistical graph of c-Fos expression in the ipsilateral and contralateral hippocampus, amygdala, LC and NTS. **F** Statistical graph of c-Fos expression in DRN. Number of samples from **B** to **E**: PTZ-vagotomy-sham group: n = 4 rats; PTZ-vagotomy-aBCI group: n = 4 rats. Mann-Whitney U-test. Data are presented as mean ± SEM.

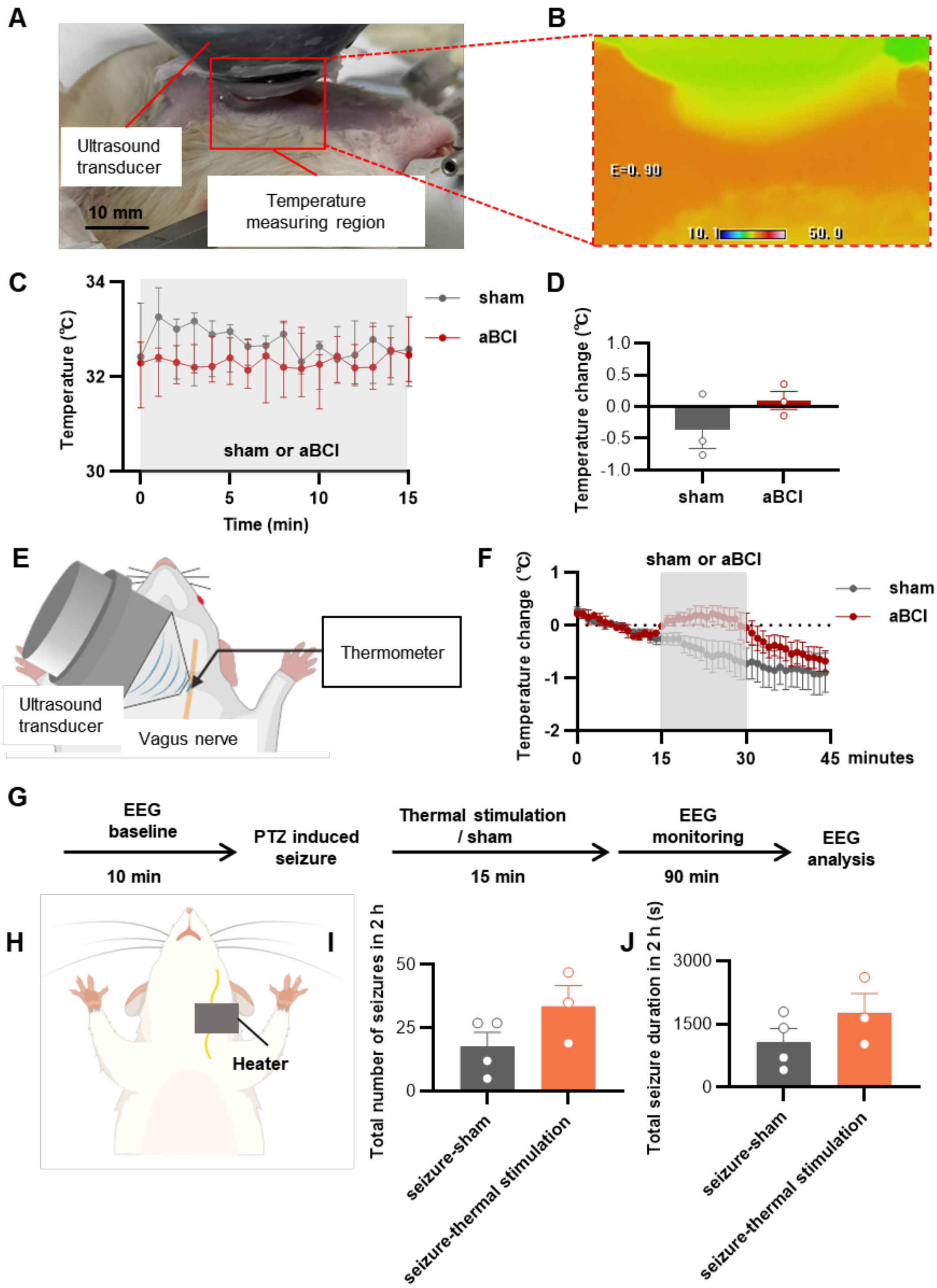


**Figure S7. The thermal effects of aBCI.**

**A** Schematic of temperature monitoring. **B** Thermography of the temperature measurement region. **C** Temperature changes during aBCI. **D** Local temperature after 15 minutes of aBCI treatment showed no significant changes compared to sham group. Sham: n = 3 rats, aBCI: n = 3 rats. **E** Schematic of temperature monitoring of the vagus

nerve. **F** Temperature changes in the vagus nerve during sham stimulation and aBCI, Sham and aBCI groups: n = 4 rats. **G** Thermal stimulation experimental procedure. **H** Schematic of thermal stimulated vagus nerve. **I** to **J** Thermal stimulation did not suppress seizures, neither in seizure number nor total duration. Sham: n = 4 rats, thermal stimulation: n = 3 rats. Mann-Whitney U-test. Data are presented as mean ± SEM.

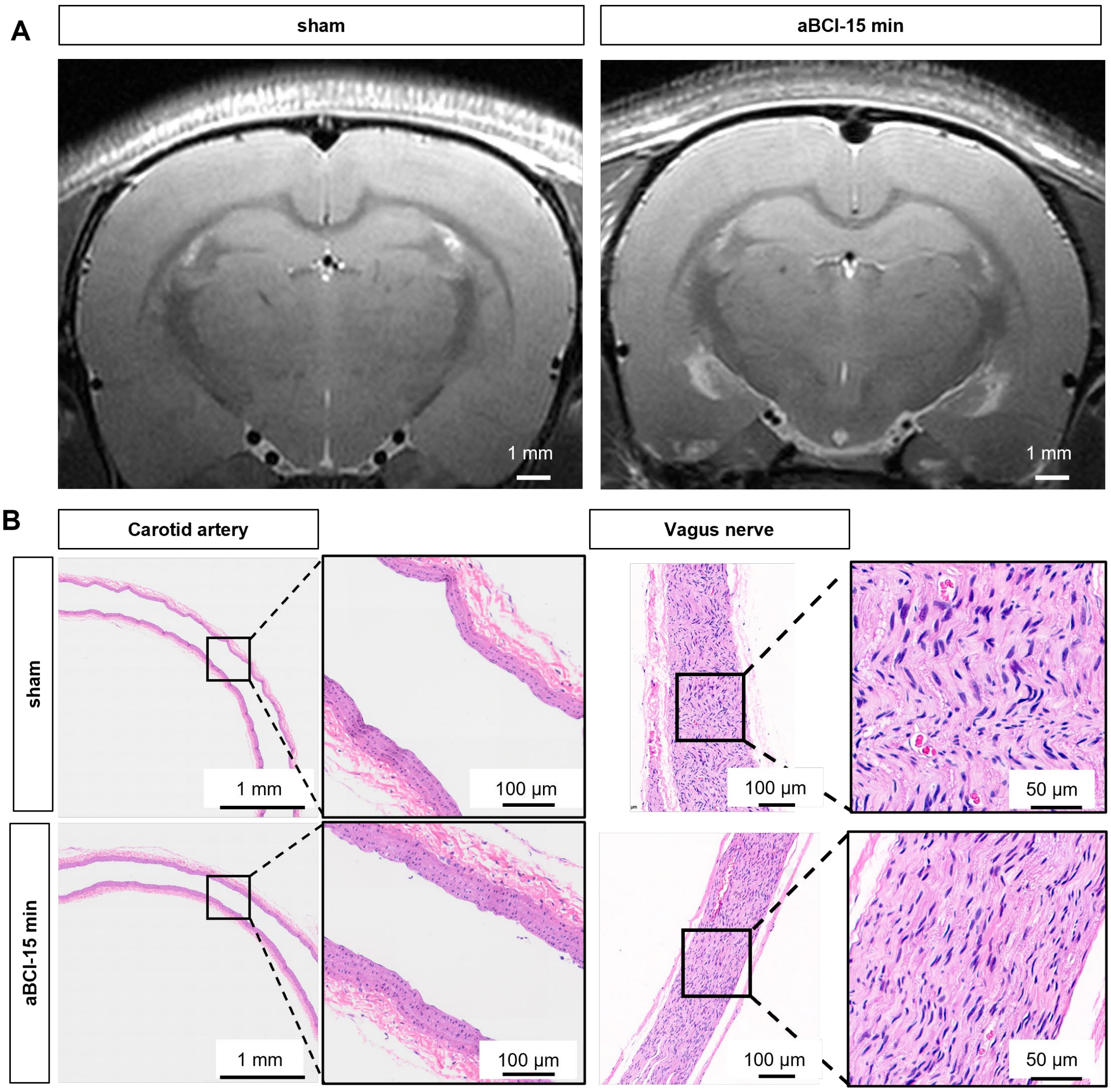


**Figure S8. Safety assessment of aBCI.**

**A** MRI of the brain. **B** H&E of the carotid artery and vagus nerve.

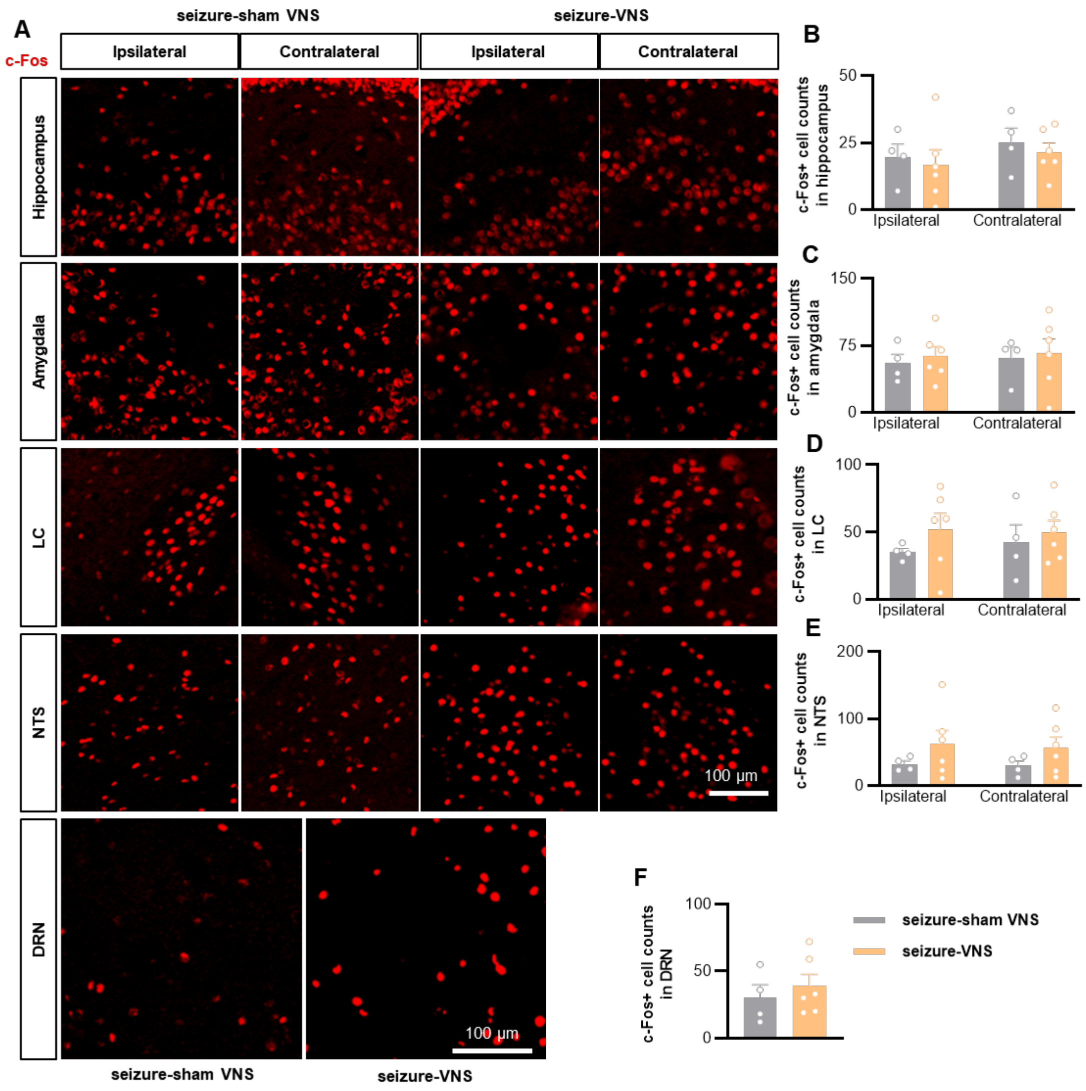


**Figure S9. Regulation of brain nuclei by the VNS.**

**A** Representative c-Fos immunofluorescence image. **B** to **E** Statistical graph of c-Fos expression in the ipsilateral and contralateral hippocampus, amygdala, LC and NTS. **F** Statistical graph of c-Fos expression in DRN. VNS upregulated c-Fos expression in the bilateral the NTS, LC, and DRN compared to the sham group, but the difference was not significant. Number of samples from **B** to **F**: sham: n = 4 rats, VNS: n = 6 rats. Mann-Whitney U-test. Data are presented as mean ± SEM.
